# Acid-Modulated Peptide Synthesis for Application on Oxide Biosensor Interfaces

**DOI:** 10.3390/nano13243092

**Published:** 2023-12-06

**Authors:** Edgar Cristóbal-Lecina, Janwa El-Maiss, Eduard Figueras, Aruna Chandra Singh, Sivashankar Krishnamoorthy, Thomas Østerbye, César Pascual García, David Andreu

**Affiliations:** 1 Proteomics and Protein Chemistry Unit, Department of Medicine and Life Sciences, Pompeu Fabra University, 08003 Barcelona, Spain; u1906229@campus.udg.edu; 2 MRT Department, Luxembourg Institute of Science and Technology, L-4420 Belvaux, Luxembourg; janwa.elmaiss@list.lu (J.E.-M.); aruna.singh@hahn-schickard.de (A.C.S.); sivashankar.krishnamoorthy@list.lu (S.K.); 3 Department of Immunology and Microbiology, University of Copenhagen, 2200 København, Denmark; thos@sund.ku.dk

**Keywords:** peptide synthesis, acid-labile protection scheme, glass surfaces, SPR chips

## Abstract

In this paper we report an acid-modulated strategy for novel peptide microarray production on biosensor interfaces. We initially selected a controlled pore glass (CPG) as a support for solid-phase peptide synthesis (SPPS) to implement a chemistry that can be performed at the interface of multiple field effect transistor (FET) sensors, eventually to generate label-free peptide microarrays for protein screening. Our chemistry uses a temporary protection of the N-terminal amino function of each amino acid building block with a tert-butyloxycarbonyl (Boc) group that can be removed after each SPPS cycle, in combination with semi-permanent protection of the side chains of trifunctional amino acid residues. Such a protection scheme with a well-proven record of application in conventional, batchwise SPPS has been fine-tuned for optimal performance on CPG and, from there, translated to SPR chips that allow layer-by-layer monitoring of amino acid coupling. Our results validate this acid-modulated synthesis as a feasible approach for producing peptides in high yields and purity on flat glass surfaces, such as those in bio-FETs.

## 1. Introduction

The semiconductor industry is currently the most active supplier of high-precision nanotechnology, thanks to the integration of field effect transistors (FETs) and metal oxide semiconductor (MOS) circuits. This has made it possible to incorporate the highest density of sensors per unit area, which presents an opportunity to address the combinatorial challenge in various omics [1]. The non-optical transduction of polymerase-assisted DNA amplification using arrays of FET pH sensors for next generation sequencing serves as an example [2]. Because peptide sequences generate many more combinatorial possibilities, the massive multiplexing capability of FET circuits could be used to create peptide microarrays to screen peptide/protein interactions, with the advantage that FET sensors can be label-free and provide, for example, information on binding kinetics. However, the requirement of implementing tailor-made functionalities for each sensor requires an approach based on in situ parallel synthesis.

Microspotting technology is currently available for the development of peptide microarrays [3,4]. However, because it is a robotic sequential technique that requires the use of pre-synthesized peptides, micro-spotting has a limited throughput and cost efficiency. Similarly, micro-drop chemistry presents challenges due to droplet evaporation, the use of delicate capillaries, and compatibility with micro-spotting ultrasound droplet generation. Other peptide microarray technologies are based on microfluidic flow chemistry that uses optically labile protecting group or in situ synthesis by laser printing of conventional fluorenylmethoxycarbonyl (Fmoc) amino acids [5,6,7,8,9]. In general, peptide microarrays generated by these methods suffer from consistency issues [10]. Furthermore, integrating these synthetic approaches into MOSFET circuits poses significant challenges at the microscale level because the three-dimensional design and heterogeneity of FETS and the microfluidics involved in the chemistry cause scattering phenomena with the UV light used in the in situ synthesis, lowering the yield and purity of the microarrays.

Egeland and Southern proposed a new method for producing microarrays by deprotection of acid labile groups using locally generated electrochemical acid [11]. After some refinements, the approach provided acceptable results with DNA microarrays [12]. For peptide synthesis, however, conditions are more demanding than for nucleotide synthesis, i.e., acidic conditions required for stepwise deprotection cycles are more extreme, and possible side reactions of amino acid side chains must be averted. We recently described an improved reactor concept for electrochemical acid production, and demonstrated that it affords good yields in the local deprotection of acid-labile groups in organic electrolytes compatible with peptide synthesis [13,14,15]. To achieve high-density microarrays, these electrochemical reactors can be miniaturized and integrated with MOS circuits. 

In tune with this innovation, we have revisited peptide synthesis protocols relying on acid-labile protection schemes, to evaluate the feasibility of obtaining high purity peptides via in situ synthesis on semiconductor oxide surfaces similar to those used in our FET biosensors [16,17]. Our goal was to validate a suitable surface capable of simulating the chemical conditions of semiconductors or metal oxides which form the interface of today’s commercial FETs. We focused on silica-based solid supports compatible with glass slide techniques as well as modern high-K dielectrics (such as TiO_2_ or HfO_2_) FETs [18,19,20]. All of these oxides have similar chemical behavior and can thus easily be derivatized by silane linkers with amino end groups (e.g., aminopropyl-triethoxysilane (APTES)) amenable to peptide synthesis [21]. 

In the initial phase of our work, precursor to a subsequent FET device, we have implemented solid-phase peptide synthesis (SPPS) on controlled pore glass (CPG) beads using acid-based protection schemes. Solid-phase synthesis refers to a well-established group of chemical technologies for biomolecule production, pioneered by Merrifield [22] for peptides (i.e., SPPS) and then extended to RNA and DNA. For readers unfamiliar with SPPS, comprehensive and authoritative reviews can be found in [23,24]. In the present work, our preliminary SPPS exercise serves a three-fold purpose: (i) to evaluate suitable combinations of orthogonally protecting groups, (ii) to define optimal coupling and deprotection conditions for product yield and purity, and (iii) to produce cleavable material for HPLC-MS monitoring of the overall viability of the process. Once the optimal synthesis conditions of the CPG were defined, we adapted them to flat surfaces that reproduce the conditions in FET sensors more closely. Specifically, we performed the synthesis on SiO_2_ surfaces layered on top of SPR chips, which allowed us to monitor an SPR signal at the stepwise incorporation of each amino acid residue. Flat glass circular (1 cm diameter) cover slides have enough surface area to produce peptide amounts amenable to HPLC-MS monitoring. 

Our results show that (i) peptides of reasonably high purity can be obtained by acid-labile (Boc-based), protecting schemes up to moderate size (>10 residues); (ii) efficient attachment of the first C-terminal amino acid ensures a successful synthetic process, and (iii) flat surfaces allow faster synthesis times, while SPR chips are useful for testing layer-by-layer additions.

## 2. Materials and Methods

### 2.1. Chemicals

The 3-(Aminopropyl)triethoxysilane (APTES), *N*,*N*-diisopropylethylamine (DIPEA), and Boc-His(Dnp)-OH were from Sigma Aldrich (Madrid, Spain). Trifluoroacetic acid (TFA) was from Carlo Erba (Sabadell, Spain). The high-purity (>99.8%) solvents, dichloromethane (DCM), *N*,*N*-dimethylformamide (DMF), and acetonitrile (MeCN), were from Fisher Scientific (Madrid, Spain). HMBA linker and 4M HCl in dioxane were purchased from Fluorochem (Hadfield, UK). In addition, 2-(1H-benzotriazol-1-yl)-1,1,3,3-tetramethyluronium hexafluorophosphate (HBTU), *N*,*N*′-diisopropylcarbodiimide (DIC), Boc-L-Trp-OH, Boc-L-Tyr-OH, Boc-L-Tyr(2-Br-Z)-OH, Boc-L-Thr-OH, Boc-Thr(tBu)-OH, Boc-L-Ser-OH, Boc-L-Ser(tBu)-OH, Boc-L-His(Trt)-OH, Boc-L-Cys-OH, and Boc-L-Met-OH were from Iris Biotech (Marktredwitz, Germany). The 4-Dimethylaminopyridine (DMAP), Boc-L-Glu(OFm)-OH, Boc-L-Asp(Ofm)-OH, and Boc-L-Cys(Fm)-OH were from Bachem (Bubendorf, Switzerland). Boc-L-Gly-OH, Boc-L-Ala-OH, Boc-L-Ile-OH, Boc-L-Pro-OH, Boc-L-Phe-OH, Boc-L-Val-OH, Boc-L-Leu-OH and Boc-L-His-OH, Boc-L-Lys(Fmoc)-OH were from Novabiochem-Merck (Madrid, Spain). All chemicals were used without further purification. 

### 2.2. Peptide Synthesis on CPG

**Figure 1:**
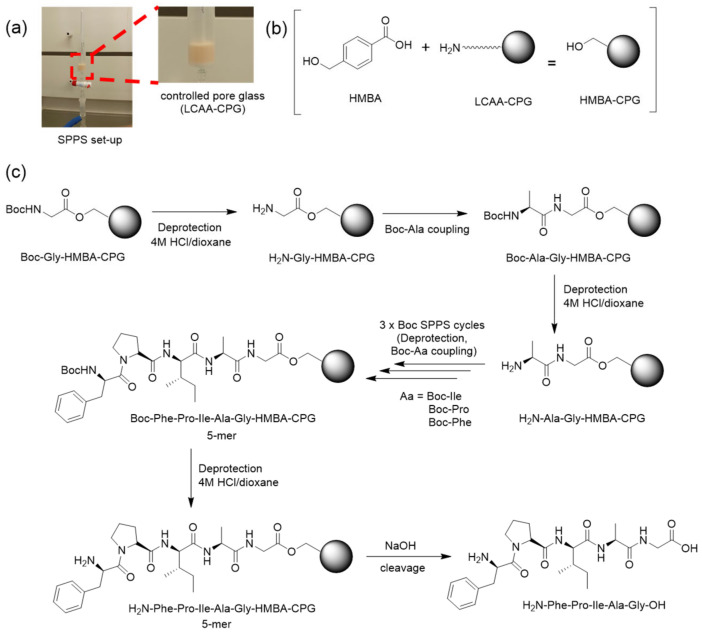
(**a**) Experimental set up for SPPS. Controlled pore glass (CPG) synthesis: filter syringe reactor containing LCAA-CPG beads and magnetic stirring bar. All synthesis steps (deprotection, coupling) took place in the system, with excess liquids (solvent, reagent) vacuum-drained into waste. (**b**) Functionalization of LCAA-CPG with HMBA linker. (**c**) Chemical steps in the synthesis of representative pentapeptide FPIAG on CPG.

SPPS was first performed manually in disposable polypropylene syringes (Figure 1a) on LCAA-CPG beads (1.2 g, 122 µmol/g). Beads were washed with DMF (×3) and DCM (×3), then the HMBA linker (186 mg, 10 eq., 1.2 mmol) and HBTU (464 mg, 10 eq., 1.2 mmol) in 4 mL DMF (0.3 M each), and DIPEA (426 µL, 20 eq., 2.4 mmol) were added and stirred for 2 h (Figure 1b). The coupling was repeated twice with intervening DCM (3×) and DMF (3×) washes. The C-terminal residue, Boc-Gly-OH (214 mg, 1.2 mmol), in 8 mL DCM (0.15 M) and DIC (95 µL, 607 µmol) were added next and vortexed for 5 min, followed by DMAP (0.1 eq., 12.22 µmol, 1.49 mg) in 100 µL DMF. The mixture was stirred for 4 h at room temperature and the coupling was repeated twice. 

The resulting Boc-Gly-HMBA-CPG (16.6 µmol), placed in a syringe reactor, was washed with DMF (3×) and DCM (3×), then Boc-deprotected with 2 × 30 min treatments with 4 M HCl/dioxane (see Table 1 for deprotection conditions). The next Boc-amino acid (10 eq., 166 µmol) was then coupled, in the presence of HBTU (10 eq., 166 µmol) and DIPEA (20 eq., 332 µmol) in 2 mL DMF, followed by DMF and DCM washes. The above deprotection–coupling cycle was repeated as required to assemble each peptide (Figure 1c; see Table 2 for sequences and further data on reaction times, conditions, etc.). 

After the chain assembly was completed, the side chain protecting groups, if any, were removed with piperidine/DMF (20:80, *v*/*v*, 2 × 10 min), followed by DMF and DCM washes, prior to cleaving the peptide from the CPG solid support. For cleavage, a 10-mg sample of peptide-HMBA-CPG beads was treated with 0.75 µL of 0.1 M NaOH/dioxane (1:3 *v*/*v*) for 2 h at RT, followed by evaporation under a N_2_ stream. The residue (CPG beads + crude peptide) was suspended in 300 µL of 10% MeCN and centrifuged. A 50-µL aliquot of the supernatant was analyzed by HPLC-MS as described below. 

Different SPPS operational parameters (e.g., deprotection conditions, coupling reagents, coupling times, Boc-amino acid concentration, stoichiometry, solvents, etc.) were investigated as summarized in Table 1 and Table 2.

### 2.3. HPLC-MS Analysis

The identity and purity of the peptides assembled on CPG was checked by HPLC-MS in an LCMS-2010 EV instrument (Shimadzu, Kyoto, Japan). For the analysis, 50 µL of a ca. 1 mg/mL solution in 10% MeCN were injected on an Aeris XB-C_18_ column (3.6 µm, 150 × 4.6 mm, Phenomenex, Torrance, CA, USA) eluted with a linear 5–95% MeCN gradient into 0.1% formic acid in H_2_O over 15 min at 1 mL/min flow rate. MS and UV detection were set at 100–2000 *m*/*z* and 220 nm, respectively. 

Peptides synthesized on glass surfaces were solubilized in 0.01% trifluoroacetic acid in water and analyzed in an UltiMate-3000 HPLC system (Thermo Fischer, Waltham, MA, USA) coupled to an LTQ/Orbitrap Elite (Thermo Fischer) high resolution mass spectrometer. For the analysis, 50 µL of peptide solution was injected on a Kinetex EVO C18 (Phenomenex) column eluted with a linear gradient of MeCN into H_2_O, both containing 0.1% formic acid.

### 2.4. Adapting SPPS to Glass Surfaces

The optimized SPPS protocols on CPG were replicated on flat glass surfaces matching the behavior of FET sensors. Glass surfaces and SiO_2_ SPR chips were functionalized as described next, and the signals recorded from SPR chips were used to monitor the progress of synthesis on the film. 

The surfaces were first cleaned with isopropanol and acetone, blow dried with N_2_ gas and activated with UV-ozone for 30 min. Then, a monolayer of 3-(aminopropyl)triethoxysilane (APTES) was grafted on the surfaces by dipping into a 5% solution of the linker in absolute ethanol and heated at 50 °C overnight. After the cleaning and functionalization of the glasses, SPPS was performed following the same protocols developed on CPG, namely (i) the incorporation of the HMBA linker; (ii) the coupling of Boc-protected amino-acid (10 eq.) in the presence of HBTU (10 eq.) and DIPEA (20 eq.), with anhydrous MeCN instead of DMF as solvent; (iii) the Boc deprotection with 50% TFA in MeCN. Further details in Figure 1c.

### 2.5. Surface Plasmon Resonance Analysis

SPR measurements were carried out in a BioNavis SPR 220A NAALI instrument (Tampere, Finland) featuring two lasers at 670 and 725 nm wavelengths. Sensor chips consisted of a SiO_2_ coating on top of a plasmonic gold layer. Chip functionalization following the above-described method was done ex situ in a chemical-resistant glass container by dipping it in a solution containing the pertinent reagents. Then, the chips were cleaned with MeCN and blow dried with N_2_. Measurements were taken after each peptide synthesis step using 10 mM PBS at 10 μL/min as flow solvent by recording shifts in the incidence angle upon analyte-surface interactions. 

## 3. Results and Discussion

### 3.1. Validation of CPG as SPPS Support

CPG was chosen to implement SPPS, prior to transfer to flat glass surfaces, as glass is similar in chemical terms to metal and semiconductor oxides. Indeed, while diffusion in CPG and flat SiO_2_ surfaces, such as those in FET biosensors, may differ, electron affinities of oxygen groups on either type of support are arguably alike, hence the outcome of CPG-based SPPS is likely to be comparable to SPPS on those other materials, in terms of product identity, yield, and purity. 

As a first step, the viability of CPG-based SPPS by the Fmoc/tBu strategy, the most widely used for current (batchwise) applications, was tested [25]. A long chain alkylamino -functionalized CPG (LCAA-CPG) support was derivatized with an Fmoc rink-amide linker [26,27] enabling attachment to the C-terminal residue and subsequent chain elongation. The linker also allowed cleavage of the peptide product from the support at any stage for detailed analytical monitoring by HPLC-MS. In this setup (Figure 1), two trial syntheses were run, (i) the tetrapeptide DKFG-amide (requiring protection at the K (Boc) and D (tBu) side chains), and (ii) the octapeptide epitope APTAPLPG-amide (no side chain protections required). In both cases, crude products of very good yield (94 and 96% by HPLC-MS, Figure S1, Supplementary Materials) were obtained, confirming CPG as a practical support for Fmoc/tBu SPPS. We were now ready for the next step, namely exploring the feasibility of CPG-SPPS by acid-controlled (de)protection strategies, in line with the aim of the project.

### 3.2. Boc-Based (Acid-Controlled) SPPS on CPG

As stated above, the ultimate goal of our project is performing SPPS on a novel platform where electrochemically generated acid is used in the repetitive deprotection cycles required for peptide chain growth. Accordingly, we explored protocols where the amino group of every residue incorporated to a peptide sequence is temporarily protected with the acid-labile tert-butyloxycarbonyl (Boc) group. This approach was pioneered by Merrifield [22] and is still in use for batchwise synthesis and specialized applications in some laboratories, albeit its somewhat demanding operational aspects have curtailed its application in favor of the now routine Fmoc/tBu strategy.

The choice of Boc as N-protecting group has in turn required to establish the viability of (i) a base-labile linker to the LCAA-CPG solid support, and (ii) base-labile protecting groups for trifunctional amino acid side chains, in order to achieve a desirable level of orthogonal protection. While base-labile linkers will not be ultimately required for sensor peptides immobilized by in situ assembly on a FET surface, in the progress towards that goal the possibility of off-line monitoring by HPLC-MS (i.e., after cleavage) the quality of peptide products is key for establishing the viability of SPPS on CPG.

Accordingly, the 4-hydroxymethylbenzoic acid (HMBA) linker [28] (Figure 1b), orthogonally cleavable with base (diluted ammonia, or 0.1 M NaOH in water/dioxane), was chosen to anchor the C-terminus of model tetrapeptide APFG to the LCAA-CPG support, and we set out to explore/optimize acid conditions for N-terminal (Boc) deprotection ensuring efficient stepwise chain growth. HPLC-MS analysis of the crude product after 0.1 M NaOH cleavage for 2 h was used to identify an optimal deprotection cocktail (acid/solvent) (Table 1). 50% TFA/DCM for 5 min, a treatment typically ensuring full Boc deprotection in batchwise SPPS, afforded only 78% of the target APFG, accompanied by deletion sequences evidencing incomplete Boc removal at each of the four deprotection cycles (93.9% on average, Table 1, entry 1). As expected, longer deprotection times ensured full Boc deprotection and a clean end product (Table 1, entry 2). 4 M HCl/dioxane, another typical deprotection agent, followed a pattern similar to TFA/DCM thus providing an alternative method (Table 1, entries 3 and 4). Solvents other than DCM and dioxane, including “green” diethyl carbonate and propylene carbonate, were tested but not found advantageous.

### 3.3. Model Boc Syntheses of FPXAG on CPG 

As a next step, and to assess the influence of the individual residue side chain structure on the viability of the process, a library of 5residue sequences was generated using the above-defined Boc chemistry protocol on HMBA-CPG and 4 M HCl/dioxane deprotection for 45 min. Sequences were based on an FPXAG model pentapeptide where four specified bifunctional amino acids flanked a variable X position, at which commercially available bifunctional (V, L, I), and trifunctional (D, E, K, N, Q, W, H, M, C, Y, T, S) residues were tested, the latter in side-chain protected form, with some exceptions (see Section 3.3.2 below). 

#### 3.3.1. Bifunctional Amino Acids at X Position

Aliphatic residues Leu, Ile and Val (L, I, V) [29], with predictably uncomplicated behavior given their bifunctional nature, were first tried at the X position of the FPXAG model peptide. FPLAG-, FPIAG- and FPVAG-HMBA-CPG were assembled as outlined above, with HBTU/DIPEA-mediated couplings (10 eq. each of Boc-amino acid and HBTU, 20 eq. DIPEA, 1 h), with final cleavage with 0.1 M NaOH/dioxane, 45 min. HPLC-MS analysis of the crude end products showed very good yields in the 94–98% range (Table 2, entries 1–3, Supplementary Materials Table S1). 

#### 3.3.2. Trifunctional Amino Acids at X Positions

Exploration of trifunctional residues for the variable position of FPXAG started with three derivatives, Asp(OFm), Glu(OFm), and Lys(Fmoc), with base-labile fluorenylmethyl (Fm)-based side chain protections. After chain assembly, and prior to cleavage from the solid support, the Fm group of FPD(OFm)AG-HMBA-CPG and the other two (E, K) peptides was removed by 20% piperidine/DMF (3 × 5 min). HPLC-MS analysis showed very good yields again (96% and 95%, respectively) for both Asp and Glu syntheses, and an acceptable 86% for Lys (Table 2, entries 4–7). As MeCN instead of DMF as coupling solvent (entry 6) caused no significant improvement, all subsequent couplings were in DMF.

The exploration was continued with amide residues Asn and Gln, for which no side chain-protected Boc-derivative was available and were thus used in unprotected form. HPLC-MS analysis of the FPQAG- and FPNAG-HMBA-CPG syntheses showed substantial differences between Asn (80%) and Gln (96%) (Table 2, entries 8–9). For Asn, significant amounts of deamidated FPDAG product were found (for further details see Table S1), not observed for Gln. Attempts to improve the Asn synthesis by modulating synthetic conditions were unsuccessful. 

Two residues with heterocycle side chains, Trp and His, were next tested in unprotected side chain version, with contrasting results. For Trp, the synthesis was very successful (96%, Table 2, entry 10), while for (unprotected) His, the target FPHAG product could not be detected (Table 2, entry 11), low solubility of Boc-His-OH most likely accounting for the failure. Alternatives to unprotected His were next sought, among which His(Dnp) and His(Trt) were most promising [30]. The former, readily incorporated (no solubility issues) into a FPH(Dnp)AG-HMBA-CPG sequence, gave 74% yield after Dnp removal (piperidine/DMF,10 × 5 min) prior to cleavage (Table 2, entry 12). The His(Trt) option gave an even more satisfactory yield (91% after Trt removal with 10% TFA/DCM and standard cleavage) (Table 2, entry 13). Interestingly, this approach may prove particularly relevant in the context of a FET platform where acid is the sole deprotection agent.

Evaluation of the X position continued with residues containing sulfur-. For Met a 76% purity was found (Table 2, entry 14), accompanied by a main byproduct of mass +16 Da readily assigned to air oxidation (synthesis under non-inert atmosphere) to the Met(O) sulfoxide. Cys, the other sulfur residue assayed as X, was predictably problematic when used unprotected (Table 2, entry 15) but practicable in its commercial Boc-Cys(Fm) version, which after piperidine/DMF deprotection and standard cleavage afforded first a modest 58% yield (Table 2, entry 16) that could, however, be improved to 75% (Table 2, entry 17) by performing the cleavage in the presence of DODT, a thiol scavenger that reduced disulfide dimer formation upon cleavage [31]. Other scavengers, such as TCEP, showed no improvements. 

The investigation of position X in FPXAG was completed with the three residues with a side chain hydroxyl group, Ser, Thr, and Tyr. All three proved viable in side chain-unprotected form (Table 2, entries 18, 20 and 22), although for Tyr the yield (56%) was rather low hence a side chain-protected version was sought [32]. The only commercially available derivative, Boc-Tyr(BrZ) with a base-labile side chain protection, provided significant improvement (95% yield after piperidine/DMF deprotection and standard acid cleavage, Table 2, entry 23). This result was not readily extendable to Ser and Thr, for which commercial side chain protected derivatives include only benzyl (disregarded, as it requires harsh anhydrous HF conditions for removal) or more acid-labile tBu, which was thus tested as only option. The results (Table 2, entries 19 and 21) were marginally improved for Ser (77 vs. 81%) but unchanged for Thr, which at 81% remains quite satisfactory. 

**Figure 2:**
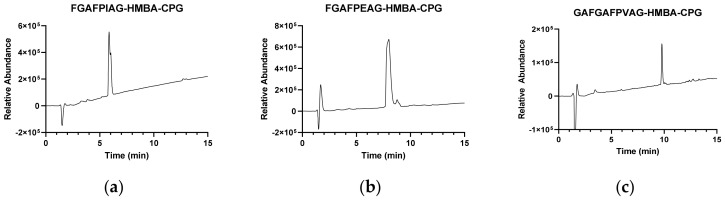
HPLC-MS analysis of octapeptide FGAFPIAG (**a**), FGAFPEAG (**b**) and decapeptide EAFGAFPEAG (**c**) crude products, representative of syntheses in Table 3.

To extend the scope and further validate the feasibility of this proof of concept, longer sequences were synthesized. First, three 8-mers (FGAFPIAG-HMBA-CPG, FGAFPVAG-HMBA-CPG, FGAFPEAG-HMBA-CPG) were synthesized by N-terminal elongation of sequences 2, 3, and 5 in Table 2 with the tripeptide FGA. In all cases, very good (91–97%) yields were achieved, including the latter sequence, with Glu(OFm) side chain-protection (Table 3, entries 1–3, and Figure 2a,b).

Elongation using two extra residues (GA and EA) did not significantly affect the quality (ca. 90%) of the product, even for EAFGAFPEAG-HMBA-CPG with two trifunctional Glu(OFm) residues (Table 3, entries 4 and 5, Figure 2c). Further extension by two (12-mer GDEAFGAFPEAG; three OFm-protected residues) or four residues (14-mer FGAFGAFGAFPVAG-HMBA-CPG) entailed a certain drop in yield but still within acceptable levels (80% and 76%, respectively; Table 3, entries 6 and 7 and Table S2). 

### 3.4. DYK Epitope Synthesis for Immunodetection

Aiming at the planned application as protein-antibody recognition platform, various sequences were synthesized with a DYK tripeptide motif specifically recognized by an anti-FLAG monoclonal antibody (clone M2) that can eventually provide a reliable readout of peptide display. 

The three trifunctional DYK residues proved challenging, a somehow predictable outcome given the modest yields found in the FPXAG library for Lys(Fmoc) or (unprotected) Tyr (Table 2, entries 7, 22). Thus, C-terminally elongated DYKG and DYKGG made with unprotected Tyr gave ca. 60% yields (Table 3, entries 8 and 10), which increased to 77% and 72%, respectively, upon BrZ protection (Table 3, entries 9 and 11). Adding one or two Asp residues at the C-terminus of DYK was reasonably tolerated (63%, entries 12 and 13) while the additional Lys in DYKK (entry 14) caused a further drop in yield, relatable to the above-mentioned performance of Lys(Fmoc).

These less-than-optimal results notwithstanding, one may reasonably argue that the >60% display levels attained for the DYK epitope on the CPG platform are likely enough to ensure antibody recognition and eventual signal amplification by immunological techniques, hence confirming the synthetic chemistry developed in this work as suitable for the intended goals of this project.

### 3.5. Translation and Optimization to Flat SiO_2_ Surfaces

**Figure 3:**
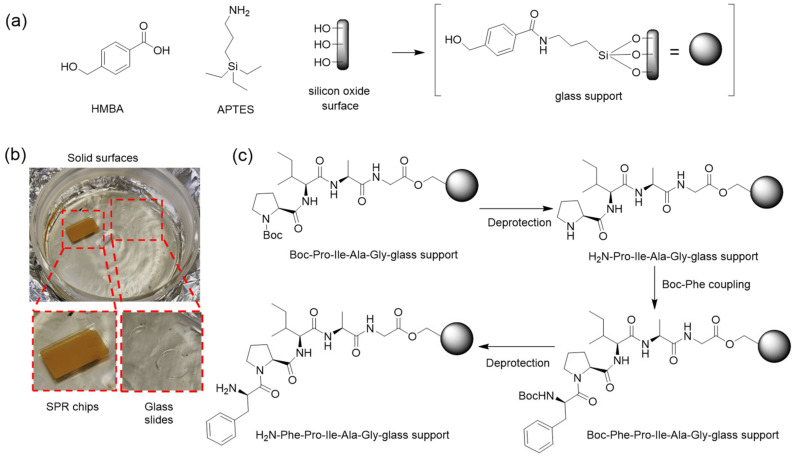
(**a**) Functionalization of glass surfaces: HMBA-APTES-SiO_2_ surface. (**b**) Adaptation to surface SPPS: SPR chips and glass slides (inset) are functionalized by dipping in the appropriate reagents/solutions. (**c**) Chemical steps in the synthesis of pentapeptide FPIAG on an APTES-functionalized SiO_2_ surface.

After successfully implementing Boc-based SPPS on LCAA-CPG beads, protocols were readily adapted to two different flat SiO_2_ surfaces, namely SPR chips and round glass slides. Figure 3a schematizes the process, involving APTES surface functionalization followed by the HMBA linker. To compare the results on each of the surfaces, we functionalized and synthesized both SPR chips and glass in the same process (Figure 3b). Figure 3c depicts the functionalization protocol equivalent to the CPG. The FPIAG 5-mer was chosen as a reference given the high yield (95%, Table 2, entry 2) obtained on the CPG and synthesis was carried out as described in Section 2.

**Figure 4:**
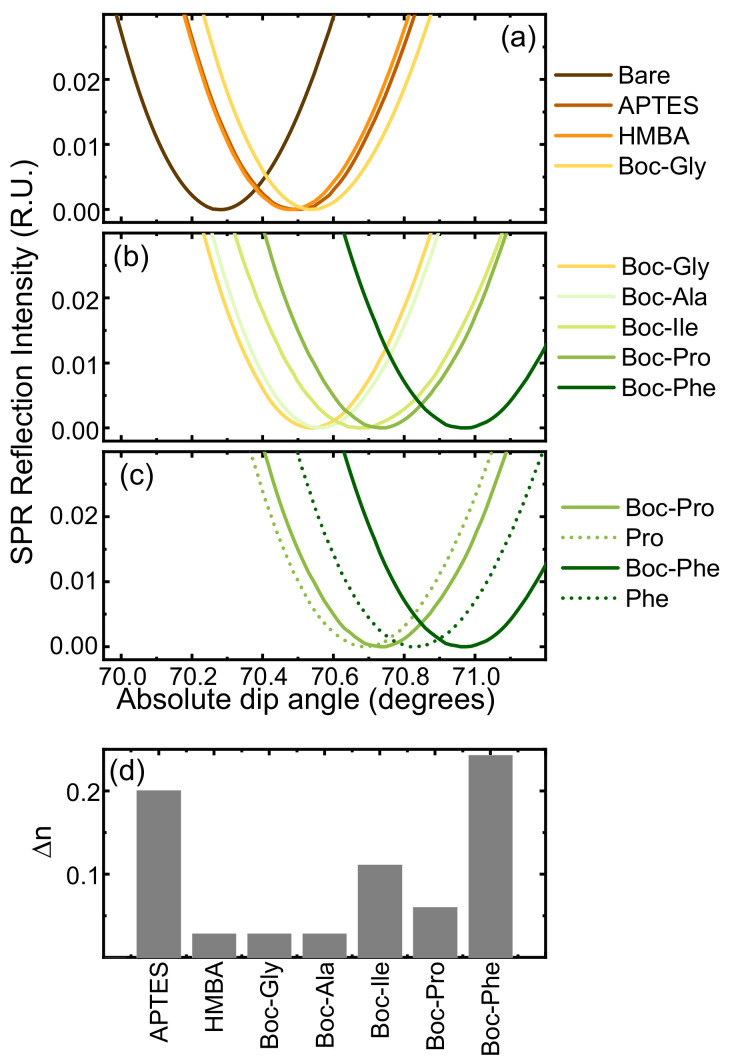
SPR monitoring of 5-mer FPIAG synthesis. (**a**) SPR dip angle change from bare surface to APTES functionalization, HMBA linker attachment and loading of first amino acid (Boc-Gly). (**b**) SPR dip angle change after Boc-amino acid addition; (**c**) SPR dip angle change illustrating amino acid addition (solid lines) and deprotection (dashed lines) steps for Pro and Phe incorporations; (**d**) Refractive index change corresponding to each layer addition.

Figure 4a–c shows the SPR monitoring of flat surface functionalization and synthesis. After each step, the SPR intensity was measured as a function of incidence angle in PBS medium. The functionalization (Figure 4a) involved grafting the SiO_2_ surface with APTES, followed by the HMBA linker and C-terminal Boc-Gly loading. Along the synthesis, layer deposition upon amino acid addition is indicated by the monotone shift of the maximum absorption angle (Figure 4b). Changes in the angle of maximum absorption measured in this medium correspond to changes in the refractive index (Figure 4d), which can be linked to variations in surface density using the De Feijter equation [33]. Maximal and minimal angle changes correspond to Boc-Phe and to Boc-Gly/Boc-Ala, as expected from their respective refractive indexes [34]. Using the side chain length of each residue (excluding protecting groups) as an estimate of volume [35] and the De Feijter equation, we found all changes to be consistent with variations in surface density of a similar order of magnitude. The surface functionalization associated with each refractive index change is estimated to be between 1 and 2 × 10^14^ molecules/cm^2^, which is well in tune with our estimate of APTES functionalization (Table S3). Figure 4c depicts the shifts before and after deprotection, as well as the decrease in refractive index absorption caused by the deprotection process, which reduces the material at the interface.

**Figure 5:**
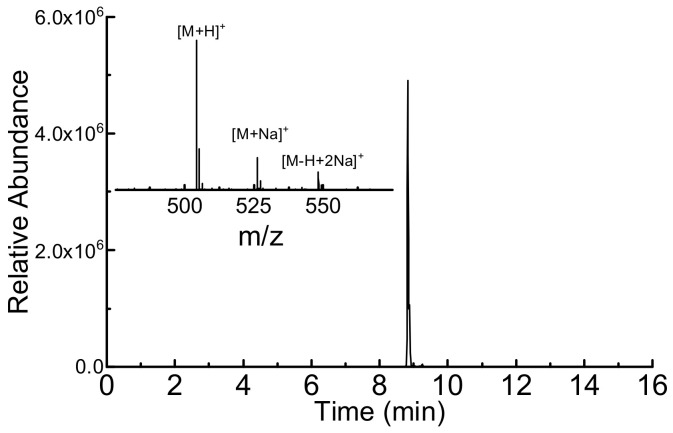
LC-MS of crude FPIAG synthesized on a SPR chip (estimated purity 86%).

The surface-bound peptides grown on the glass slides as well as SPR chips were cleaved and analyzed by LC-MS. Results show rather good yield (94%) on glass, similar to those found earlier on CPG (Table 2, entry 2) and slightly lower on SPR chips (86%, Figure 5), altogether corroborating the reproducibility of Boc-based SPPS on various surfaces (porous and flat) and paving the way for eventual transfer to FET platforms. 

As flat SiO_2_ surfaces have significantly less limitations regarding diffusion relative to CPG, we explored accelerating the synthesis by decreasing the reaction times about one order of magnitude relative to standard SPPS (Table 4). 

**Figure 6:**
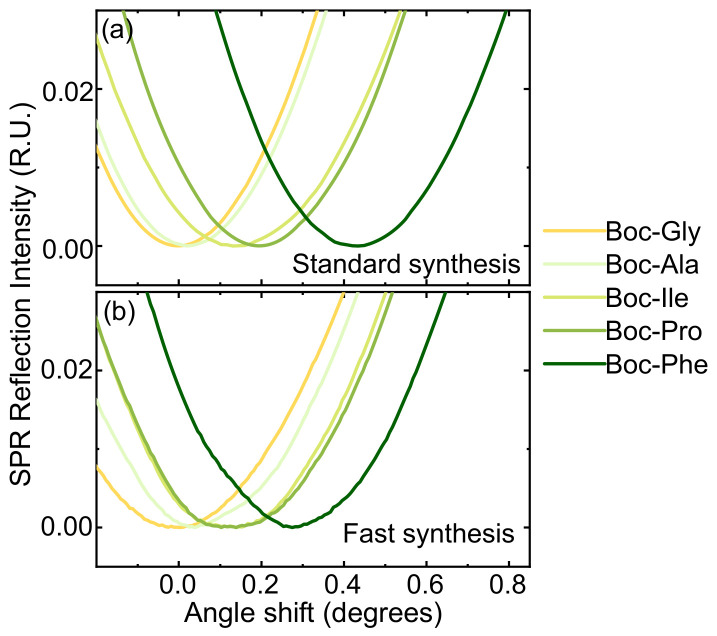
Comparison of changes in refractive angle during the standard (**a**) and fast (**b**) syntheses of FPIAG.

To ensure proper anchoring, the C-terminal Gly of the test FPIAG sequence was coupled thrice and coupling times were kept to 2 × 5 min, with a single Boc deprotection step (50% TFA, 5 min). Figure 6 shows the SPR data comparing both the standard and the fast synthesis. Both changes in the refractive index show a similar trend, although an overall smaller change in the fast synthesis is observed, which we attribute to less saturation of the surface. The synthesis time was reduced ~5-fold from ca. 20 to 4 h, (Figure 6), while the MS analysis (Figure S2), also validates the process with no detriment to product quality (ca. 90% yield). 

## 4. Conclusions

In this work we have used a controlled pore glass (CPG) solid support as a demonstration stage for SPPS on glass surfaces using acid-labile protection schemes. A 5-mer model peptide (FPXAG), with different residues at the X position, served to evaluate the feasibility of the method, which was extended to longer peptides, as well as to peptides containing DYK motifs suitable for antibody recognition. Overall, with isolated exceptions, the SPPS methodology developed for CPG proved quite robust and versatile, leading to high purity crude products and thus providing a valid proof of concept. The CPG results have been extended straightforwardly to flat glass SiO_2_ surfaces such as those of SPR chips, with good reproducibility and the additional advantage of SPR monitoring of the synthetic process. As flat surfaces have fewer limitations than CPG in regard to the diffusion of chemicals, the experimental conditions were adapted for the faster synthesis protocols with no significant impact on product purity. In conclusion, the approach described here paves the way for biosensor/FET-based peptide synthesis on semiconductor oxide platforms such as SiO_2_ that can act as microarray devices for the detection of peptide–protein interactions. A technology combining electrochemical acid production in a microfluidic set-up with in situ peptide synthesis allows one to envision promising applications for peptide-antigen interactions, proteomics, and personalized medicine. 

## Figures and Tables

**Table 1:** Boc deprotection conditions tested.

Entry	Acid	Solvent	Time	Boc Removal *
1	50% TFA	DCM	5 min	93.9% (78%)
2	50% TFA	DCM	25 min	100% (100%)
3	4 M HCl/dioxane	dioxane	5 min	96.8% (88%)
4	4 M HCl/dioxane	dioxane	25 min	100% (100%)

* Average per cycle; estimated from the HPLC purity of APFG test peptide (in parenthesis).

**Table 2:** Model peptide FPXAG syntheses with varying X residues.

Entry	Sequence	Side Chain Protection ^a^	Solvent ^b^	Time ^b^	Deprotection Time ^c^	Yield ^d^
1	FPLAG	none	DMF	1 h (×2)	45 min (×2)	98%
2	FPIAG	none	DMF	1 h (×2)	45 min (×2)	95%
3	FPVAG	none	DMF	1 h (×2)	45 min (×2)	94%
4	FPDAG	OFm	DMF	1 h (×2)	45 min (×2)	96%
5	FPEAG	OFm	DMF	1 h (×2)	45 min (×2)	95%
6	FPEAG	OFm	MeCN	1 h (×2)	45 min (×2)	95%
7	FPKAG	Fmoc	DMF	1 h (×2)	45 min (×2)	86%
8	FPNAG	none	DMF	30 min (×2)	30 min	80%
9	FPQAG	none	DMF	30 min (×2)	30 min	96%
10	FPWAG	none	DMF	30 min (×2)	30 min	96%
11	FPHAG	none	DMF	30 min (×2)	30 min	0%
12	FPHAG	Dnp	DMF	30 min (×2)	30 min (×2)	74%
13	FPHAG	Trt	DMF	30 min (×2)	3 h	91%
14	FPMAG	none	DMF	30 min (×2)	30 min	76%
15	FPCAG	none	DMF	30 min (×2)	30 min	0%
16	FPCAG	Fm	DMF	30 min (×2)	30 min	58%
17	FPCAG	Fm/DODt	DMF	30 min (×2)	30 min	75%
18	FPSAG	none	DMF	25 min (×2)	45 min	77%
19	FPSAG	tBu	DMF	25 min (×2)	45 min	82%
20	FPTAG	none	DMF	25 min (×2)	45 min	81%
21	FPTAG	tBu	DMF	25 min (×2)	45 min	81%
22	FPYAG	none	DMF	25 min (×2)	45 min	56%
23	FPYAG	BrZ	DMF	25 min (×2)	45 min	95%

^a^ Trifunctional X residues in the middle of the sequence. ^b^ Coupling reaction solvent and time. All couplings were performed with 10 eq. each of Boc-amino acid and HBTU (10 eq.), in the presence of DIPEA (20 eq.). ^c^ Boc N-terminal deprotection with 4 M HCl/dioxane. Different deprotection times reflect insights learned along library synthesis. ^d^ Percentage of target FPXAG-acid in the crude end product found by HPLC-MS.

**Table 3:** Longer sequences and DYK-containing peptides.

Entry	Sequence	Side Chain Protection	Coupling Time ^a^	Yield ^b^
1	FGAFPIAG	none	30 min (×2)	94%
2	FGAFPVAG	none	30 min (×2)	91%
3	FGAFPEAG	OFm	30 min (×2)	97%
4	GAFGAFPVAG	none	30 min (×2)	90%
5	EAFGAFPEAG	OFm × 2	25 min (×2)	89%
6	GDEAFGAFPEAG	OFm × 3	25 min (×2)	80%
7	FGAFGAFGAFPIAG	none	25 min (×2)	76%
8	DYKG	OFm, none and Fmoc	25 min (×2)	60%
9	DYKG	OFm, 2-Br-Z and Fmoc	25 min (×2)	77%
10	DYKGG	OFm, none and Fmoc	25 min (×2)	62%
11	DYKGG	OFm, 2-Br-Z and Fmoc	25 min (×2)	72%
12	DYKD	OFm, none, Fmoc and OFm	25 min (×2)	63%
13	DYKDD	OFm, none, Fmoc and OFm (×2)	25 min (×2)	63%
14	DYKK	OFm, none, Fmoc (×3)	25 min (×2)	57%

^a^ All couplings were performed with 10 eq. each of Boc-amino acid and HBTU (10 eq.), in the presence of DIPEA (20 eq.), with DMF as solvent. ^b^ % of target peptide in the crude end product found by HPLC-MS.

**Table 4:** Reaction times of standard vs. fast synthesis.

	APTES Functionalization	HMBA-Linker Attachment	C-Terminal Anchoring	AA Coupling	Deprotection
Standard	12 h	120 min	5 h	60 min	30 min
Fast	3 h	5 min	3 × 5 min	2 × 5 min	5 min

## Data Availability

The datasets used or analyzed during the current study are available from the corresponding author upon reasonably request.

## Supplementary Materials

The following supporting information can be downloaded at: https://www.mdpi.com/article/10.3390/nano13243092/s1, Figure S1: Structure, HPLC and MS data of APTAPLPG (Fmoc synthesis). Figure S2: LC-MS of standard and fast FPIAG syntheses on-glass surface. Table S1: Sequences, synthetic and analytical data of FPXAG peptide series. Table S2: Sequences, synthetic and analytical data of longer and DYK-based peptide series. Table S3: Parameters used for the De Feijter equation.

## References

[1] Syu Y.-C., Hsu W.-E., Lin C.-T. (2018). Review—Field-Effect Transistor Biosensing: Devices and Clinical Applications. ECS J. Solid State Sci. Technol..

[2] Rothberg J.M., Hinz W., Rearick T.M., Schultz J., Mileski W., Davey M., Leamon J.H., Johnson K., Milgrew M.J., Edwards M. (2011). An Integrated Semiconductor Device Enabling Non-Optical Genome Sequencing. Nature.

[3] Beyer M., Felgenhauer T., Ralf Bischoff F., Breitling F., Stadler V. (2006). A Novel Glass Slide-Based Peptide Array Support with High Functionality Resisting Non-Specific Protein Adsorption. Biomaterials.

[4] Li J., Carney R.P., Liu R., Fan J., Zhao S., Chen Y., Lam K.S., Pan T. (2018). Microfluidic Print-to-Synthesis Platform for Efficient Preparation and Screening of Combinatorial Peptide Microarrays. Anal. Chem..

[5] Simon M.D., Heider P.L., Adamo A., Vinogradov A.A., Mong S.K., Li X., Berger T., Policarpo R.L., Zhang C., Zou Y. (2014). Rapid Flow-Based Peptide Synthesis. ChemBioChem.

[6] Truex N.L., Holden R.L., Wang B.Y., Chen P.G., Hanna S., Hu Z., Shetty K., Olive O., Neuberg D., Hacohen N. (2020). Automated Flow Synthesis of Tumor Neoantigen Peptides for Personalized Immunotherapy. Sci. Rep..

[7] Hartrampf N., Saebi A., Poskus M., Gates Z.P., Callahan A.J., Cowfer A.E., Hanna S., Antilla S., Schissel C.K., Quartararo A.J. (2020). Synthesis of Proteins by Automated Flow Chemistry. Science.

[8] Mijalis A.J., Thomas D.A., Simon M.D., Adamo A., Beaumont R., Jensen K.F., Pentelute B.L. (2017). A Fully Automated Flow-Based Approach for Accelerated Peptide Synthesis. Nat. Chem. Biol..

[9] Welden R., Schöning M.J., Wagner P.H., Wagner T. (2020). Light-Addressable Electrodes for Dynamic and Flexible Addressing of Biological Systems and Electrochemical Reactions. Sensors.

[10] Price J.V., Tangsombatvisit S., Xu G., Yu J., Levy D., Baechler E.C., Gozani O., Varma M., Utz P.J., Liu C.L. (2012). On Silico Peptide Microarrays for High-Resolution Mapping of Antibody Epitopes and Diverse Protein-Protein Interactions. Nat. Med..

[11] Egeland R.D., Southern E.M. (2005). Electrochemically Directed Synthesis of Oligonucleotides for DNA Microarray Fabrication. Nucleic Acids Res..

[12] Maurer K., Cooper J., Caraballo M., Crye J., Suciu D., Ghindilis A., Leonetti J.A., Wang W., Rossi F.M., Stöver A.G. (2006). Electrochemically Generated Acid and Its Containment to 100 Micron Reaction Areas for the Production of DNA Microarrays. PLoS ONE.

[13] Balakrishnan D., Lamblin G., Thomann J.S., Van Den Berg A., Olthuis W., Pascual-García C. (2018). Electrochemical Control of PH in Nanoliter Volumes. Nano Lett..

[14] El Maiss J., Balakrishnan D., Pascual C. (2022). Universal Control of Protons Concentration Using Electrochemically Generated Acid Compatible with Miniaturization. Nanoscale Adv..

[15] Balakrishnan D., El Maiss J., Olthuis W., Pascual García C. (2023). Miniaturized Control of Acidity in Multiplexed Microreactors. ACS Omega.

[16] Shukla R.P., Bomer J.G., Wijnperle D., Kumar N., Georgiev V.P., Singh A.C., Krishnamoorthy S., Pascual García C., Pud S., Olthuis W. (2022). Planar Junctionless Field-Effect Transistor for Detecting Biomolecular Interactions. Sensors.

[17] Rollo S., Rani D., Leturcq R., Olthuis W., Pascual García C. (2019). A High Aspect Ratio Fin-Ion Sensitive Field Effect Transistor: Compromises towards Better Electrochemical Bio-Sensing. Nano Lett..

[18] Walsh M.K., Wang X., Weimer B.C. (2001). Optimizing the Immobilization of Single-Stranded DNA onto Glass Beads. J. Biochem. Biophys. Methods.

[19] Sheng H., Ye B.C. (2009). Different Strategies of Covalent Attachment of Oligonucleotide Probe onto Glass Beads and the Hybridization Properties. Appl. Biochem. Biotechnol..

[20] Rollo S., Rani D., Olthuis W., Pascual García C. (2020). High Performance Fin-FET Electrochemical Sensor with High-k Dielectric Materials. Sens. Actuators B Chem..

[21] Rani D., Rollo S., Olthuis W., Krishnamoorthy S., García C.P. (2021). Combining Chemical Functionalization and Finfet Geometry for Field Effect Sensors as Accessible Technology to Optimize PH Sensing. Chemosensors.

[22] Merrifield R.B. (1963). Solid Phase Peptide Synthesis. I. The Synthesis of a Tetrapeptide. J. Am. Chem. Soc..

[23] Barany G., Merrifield R.B., Gross E., Meienhofer J. (1979). Solid Phase Peptide Synthesis. The Peptides.

[24] Fields G.B., Noble R.L. (1990). Solid Phase Peptide Synthesis Utilizing 9-fluorenylmethoxycarbonyl Amino Acids. Int. J. Pept. Protein Res..

[25] Liu Y., Wang X.F., Chen Y., Zhang L.H., Yang Z.J. (2012). A Solid-Phase Method for Peptide-SiRNA Covalent Conjugates Based on Click Chemistry. MedChemComm.

[26] Rink H. (1987). Solid-Phase Synthesis of Protected Peptide Fragments Using a Trialkoxy-Diphenyl-Methylester Resin. Tetrahedron Lett..

[27] Bernatowicz M.S., Daniels S.B., Köster H. (1989). A Comparison of Acid Labile Linkage Agents for the Synthesis of Peptide C-Terminal Amides. Tetrahedron Lett..

[28] Hansen J., Diness F., Meldal M. (2016). C-Terminally Modified Peptides via Cleavage of the HMBA Linker by O-, N- or S-Nucleophiles. Org. Biomol. Chem..

[29] In this work the IUPAC-IUB one- and three-letter codes for amino acid residues (Pure & Appl. Chem., Vol. 56, No. 5, pp. 595–624, 1984) are used, though not strictly interchangeably: To denote individual residues (e.g., Leu), the three letter code is preferred; for longer sequences, the shorter, one-letter notation is adopted.

[30] Geiger R., König W., Gross E., Meienhofer J. (1981). Amine Protecting Groups. The Peptides.

[31] Mthembu S.N., Sharma A., Albericio F., de la Torre B.G. (2020). Breaking a Couple: Disulfide Reducing Agents. ChemBioChem.

[32] Yamashiro D., Li C.H. (1973). Protection of Tyrosine in Solid Phase Peptide Synthesis. J. Org. Chem..

[33] De Feijter J.A., Benjamins J., Veer F.A. (1978). Ellipsometry as a Tool to Study the Adsorption Behavior of Synthetic and Biopolymers at the Air–Water Interface. Biopolymers.

[34] Zhao H., Brown P.H., Schuck P. (2011). On the Distribution of Protein Refractive Index Increments. Biophys. J..

[35] Harpaz Y., Gerstein M., Chothia C. (1994). Volume Changes on Protein Folding. Structure.

