# Supplementary material for: Acid-Modulated Peptide Synthesis for Application on Oxide Biosensor Interfaces
Source: Nanomaterials (Basel). 2023 Dec 6;13(24):3092. doi: 10.3390/nano13243092 (PMC10746054; doi:10.3390/nano13243092)
Supplement: Supplementary file 1 [file nanomaterials-13-03092-s001.zip › nanomaterials-2739675-supplementary.pdf]

Supplementary information

Acid-modulated peptide synthesis for application on oxide biosensor interfaces

Edgar Cristóbal-Lecina [1,*] Janwa El-Maiss[2], Eduard Figueras[1], Aruna Chandra Singh[2], Sivashankar Krishnamoorthy[2], Thomas Østerbye[3], César Pascual García [2,*] and David Andreu [1,*]

1 Proteomics and Protein chemistry Unit, Department of Medicine and Life Sciences, Pompeu Fabra University, 08003 Barcelona, Spain; edgar.cristobal@upf.edu; david.andreu@upf.edu
2 Luxembourg insitute of science and technology, MRT department, l-4420 Belvaux, Luxembourg
3 Department of Immunology and Microbiology, University of Copenhaguen, Denmark, thos@sund.ku.dk

* Correspondence: cesar.pascual@list.lu; david.andreu@upf.edu, edgar.cristobal@upf.edu.

Contents

	Page
• Figure S1: Structure, HPLC and MS data of APTAPLPG (Fmoc synthesis)	S3
• Table S1: Sequences, synthetic and analytical data of FPXAG peptide series	S4-S11
• Table S2: Sequences, synthetic and analytical data of longer and DYK-based peptide series	S12-S16
• Figure S2: LC-MS of standard and fast FPIAG syntheses on-glass surface	S17
• Table S3: Parameters used for the De Feijter equation	S18

Figure S1

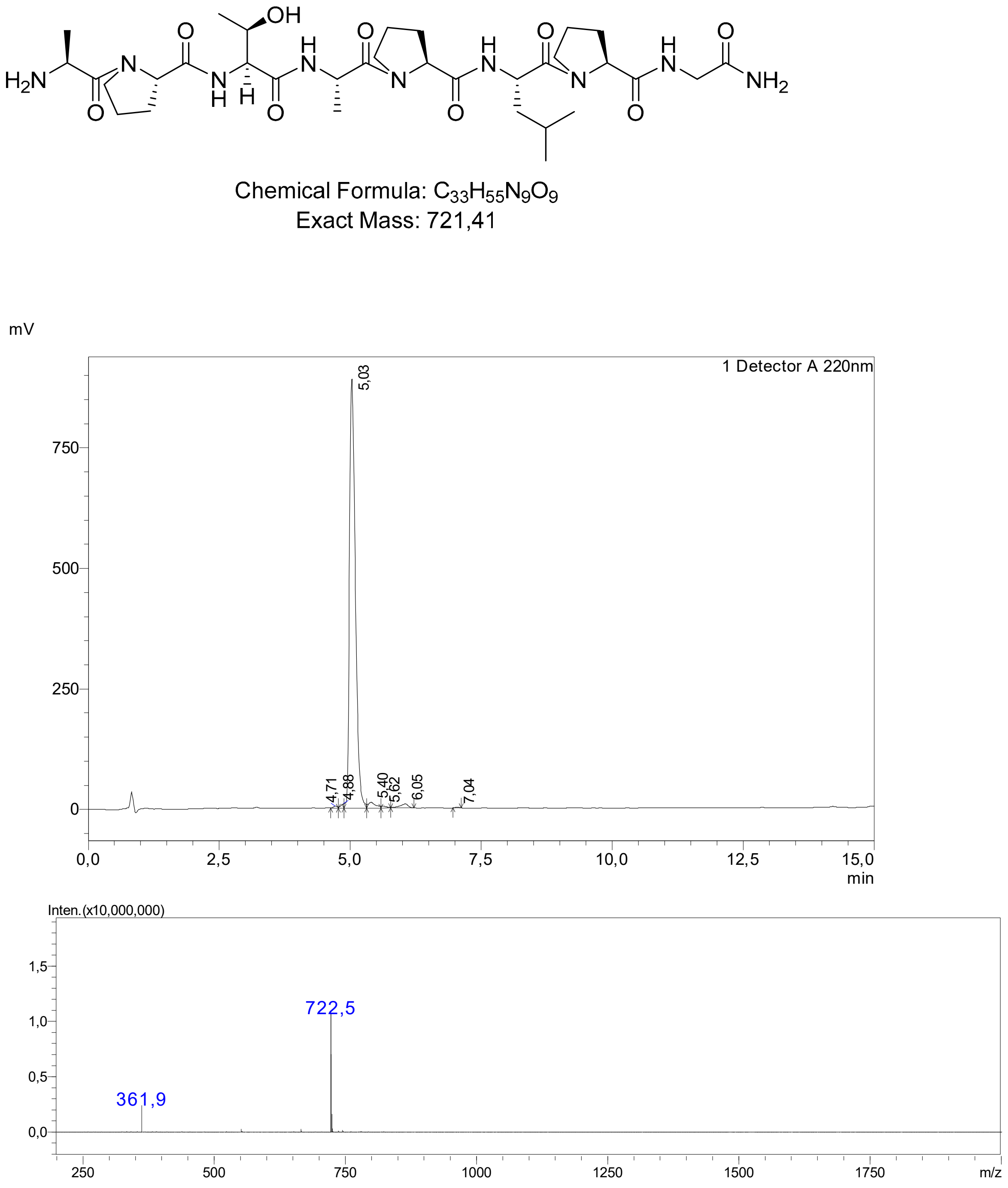
OH
H₂N
N
NH
H
O
NH2
Chemical Formula: $C_{33}H_{55}N_9O_9$
Exact Mass: 721,41
mV
1 Detector A 220nm
5,03
4,71
4,88
5,40
5,62
6,05
7,04
750
500
250
0
0,0
2,5
5,0
7,5
10,0
12,5
15,0
min
Inten.(x10,000,000)
1,5
1,0
0,5
0,0
722,5
361,9
250
500
750
1000
1250
1500
1750
m/z

Table S1

Entry	Peptides[1]	Protecting group	Theoretical Mass (Da)	Experimental Mas (Da)[2]	HPLC retention time (min)	Gradient[3]	Purity[4]
1	FPLAG	None	503.26	504.19	6.8	5-40%[a]	98%
2	FPIAG	None	503.26	504.30	6.5	5-40%[a]	95%
3	FPVAG	None	489.25	490.20	6.0	5-40%[a]	94%
4	FPDAG	OFm	505.21	506.12	4.2	5-40%[a]	96%
5	FPEAG	OFm	519.22	520.25	4.2	5-40%[a]	95%
6	FPEAG[5]	OFm	519.22	520.25	4.2	5-40%[a]	95%
7	FPKAG	Fmoc	518.27	519.30	5.0	5-40%[a]	86%
8	FPNAG	None	504.22	505.25	4.5	5-40%[a]	80%
9	FPQAG	None	518.24	519.25	4.5	5-40%[a]	96%
10	FPWAG	None	576.26	577.30	8.9	5-40%[a]	96%
11	FPHAG	None	527.24	n.d.	-	-	-
12	FPHAG	Dnp	527.24	528.10	0.4	5-95%[b]	74%
13	FPHAG	Trt	527.24	528.31	3.0	5-40%[a]	91%
14	FPMAG	None	521.22	522.20	4.2	5-40%[a]	76%
15	FPCAG	None	493.19	n.d.	-	-	-
16	FPCAG	Fm	493.19	493.55	1.2	5-95%[b]	58%
17	FPCAG	Fm/DODT	493.19	493.35	1.2	5-95%[b]	75%
18	FPSAG	None	477.21	478.20	4.0	5-40%[a]	77%
19	FPSAG	tBu	477.21	478.33	4.0	5-40%[a]	82%
20	FPTAG	None	491.23	492.25	4.0	5-40%[a]	81%
21	FPTAG[6]	tBu	491.23	492.39	4.0	5-40%[a]	81%
22	FPYAG	None	553.24	554.30	6.0	5-40%[a]	56%
23	FPYAG	2-Br-Z	553.24	554.34	6.0	5-40%[a]	95%

[1] All peptides are C-terminal carboxylic acid; [2] Determined by LC-MS; [3] Linear MeCN (+0.036% TFA) into H_2O (+0.045% TFA) over 15 min (a) or 5 min (b); [4] Determined by analytical HPLC. n.d : not determined.

1-FPLAG

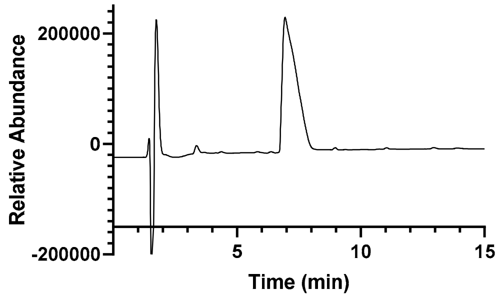

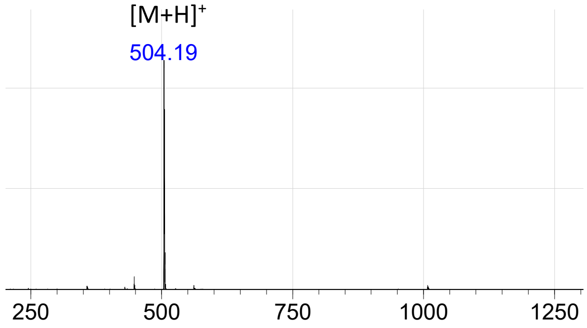

2-FPIAG

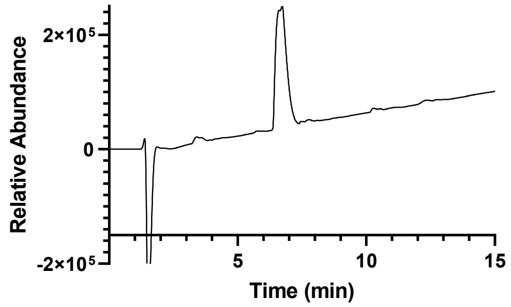

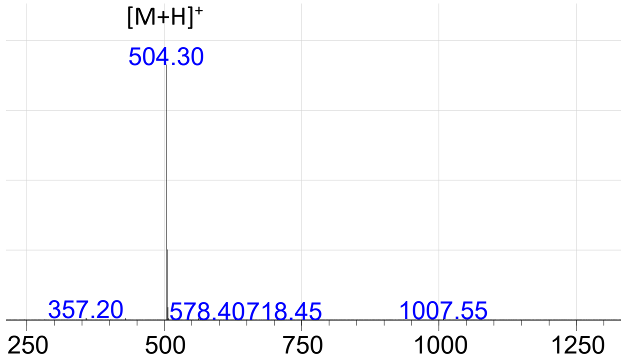

3-FPVAG

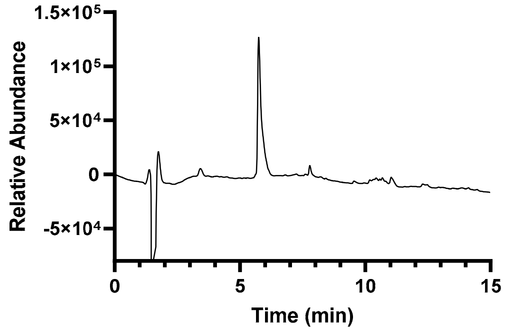

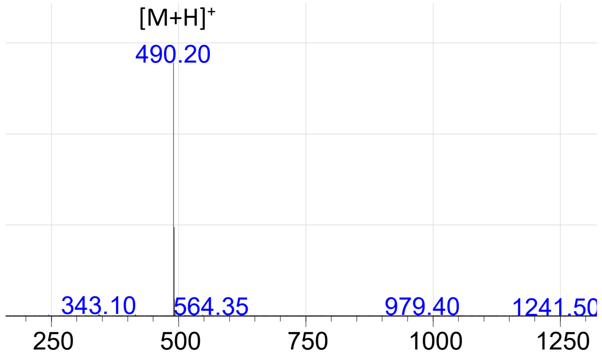

4-FPDAG

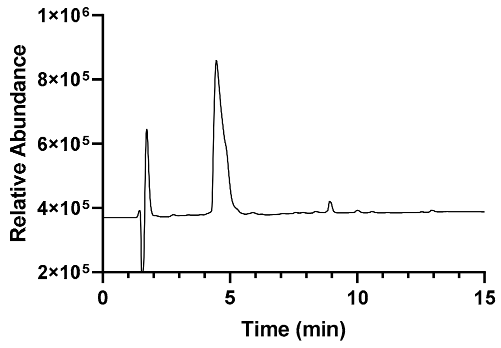

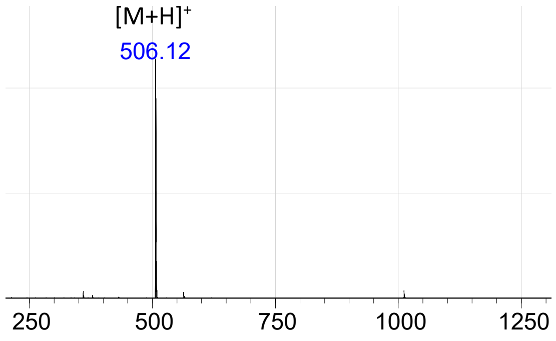

5-FPEAG

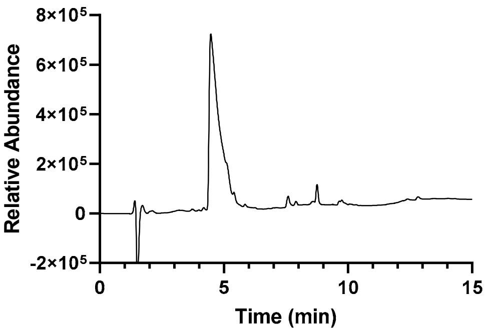

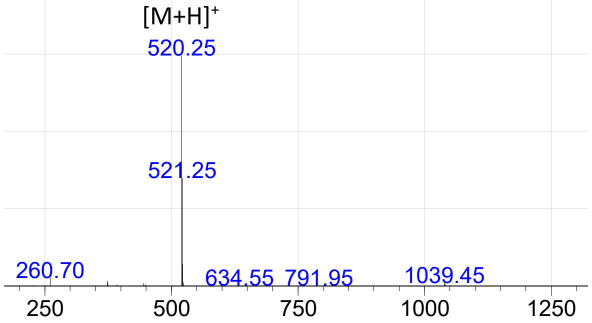

6-FPEAG

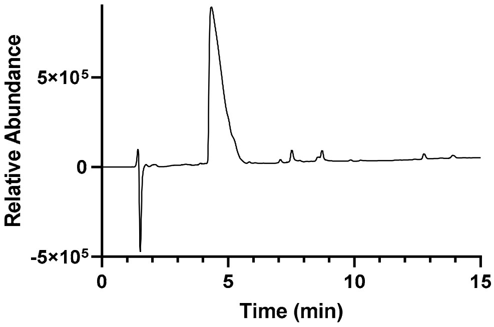

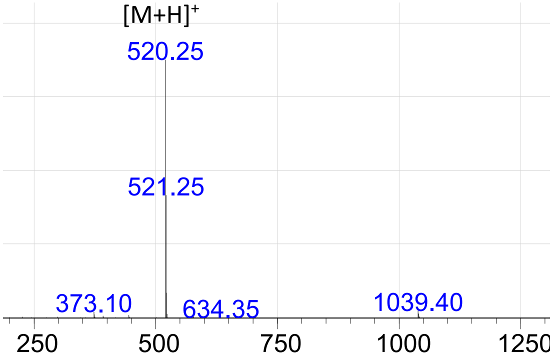

7-FPKAG

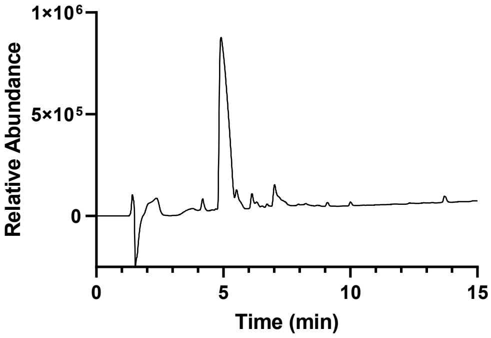

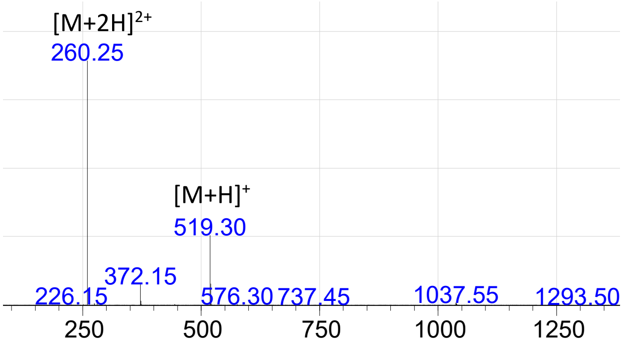

8-FPNAG

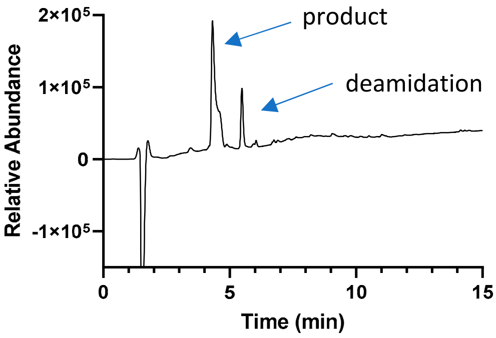

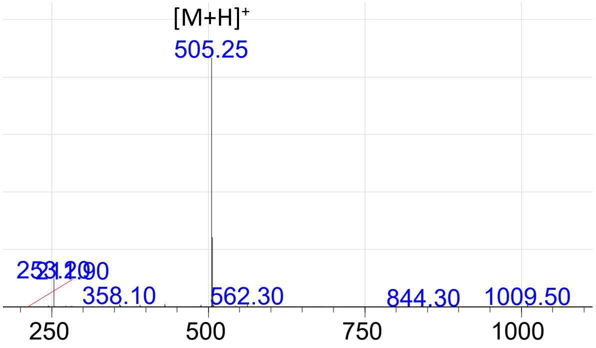

9-FPQAG

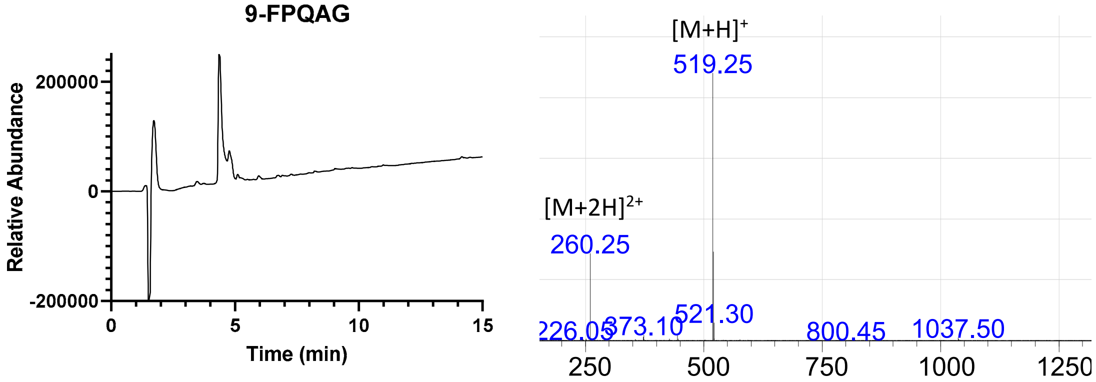

10-FPWAG

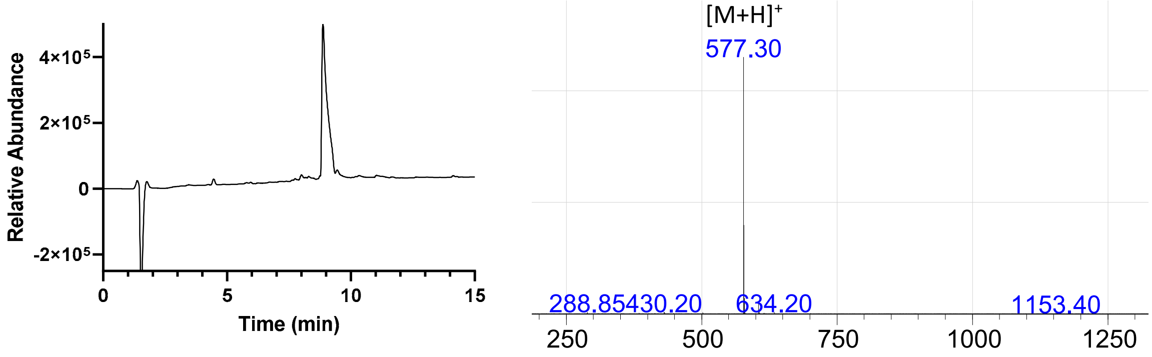

11-FPHAG

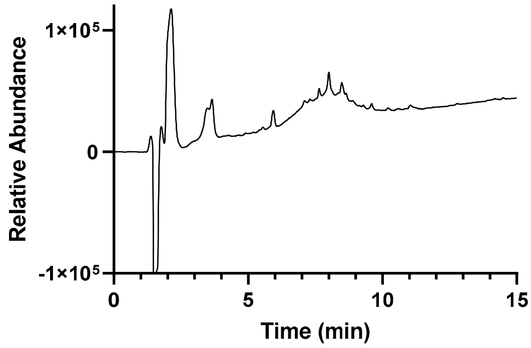

No mass compatible with 11 found

12-FPHAG

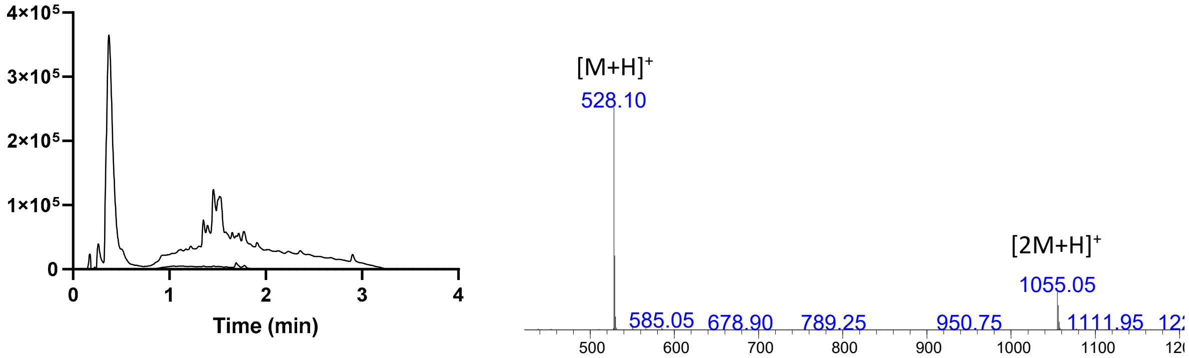

13-FPHAG

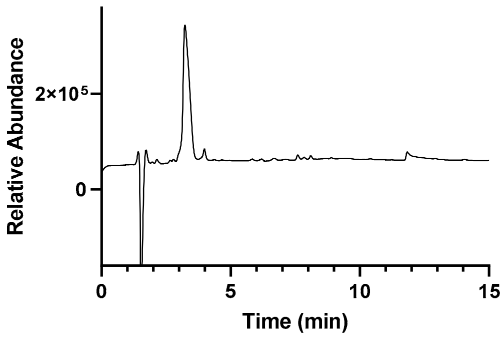

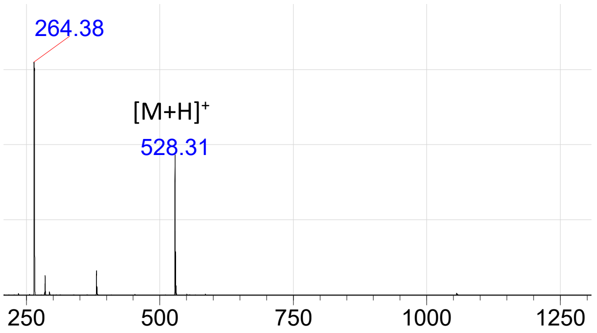

14-FPMAG

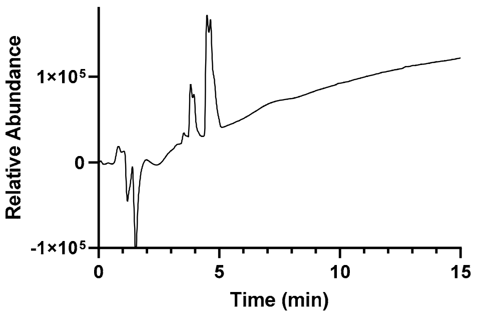

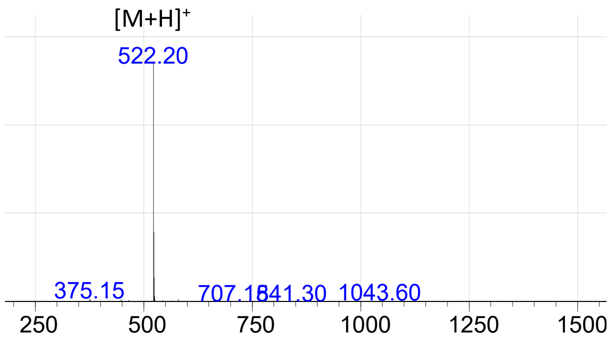

16-FPCAG

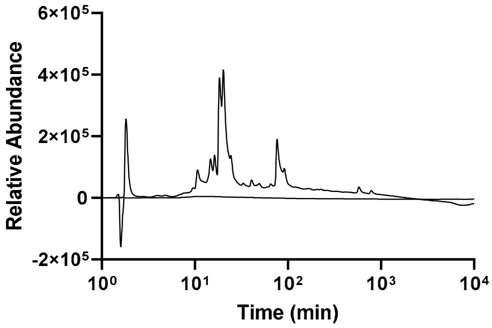

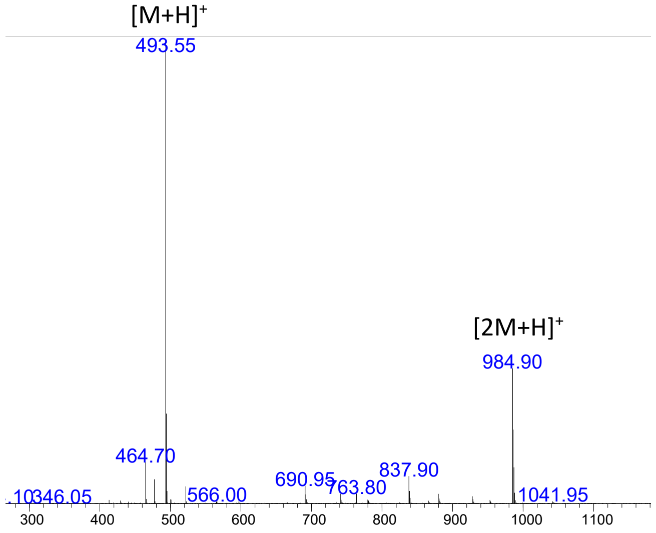

17-FPCAG

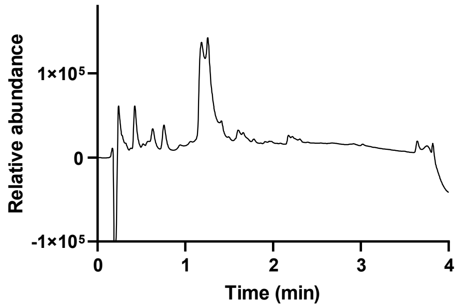

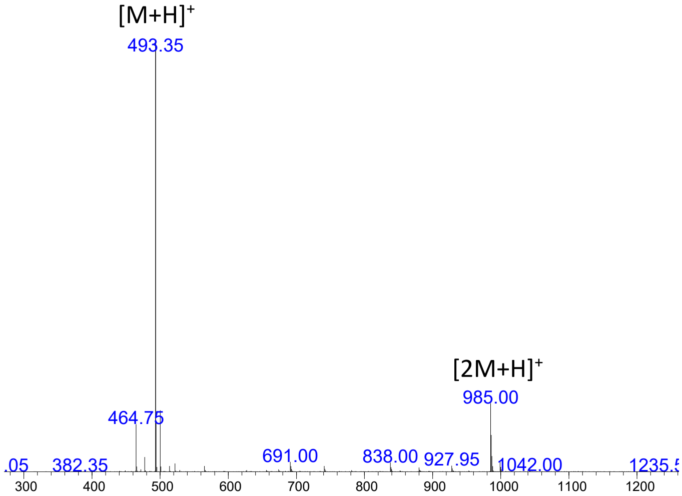

18-FPSAG

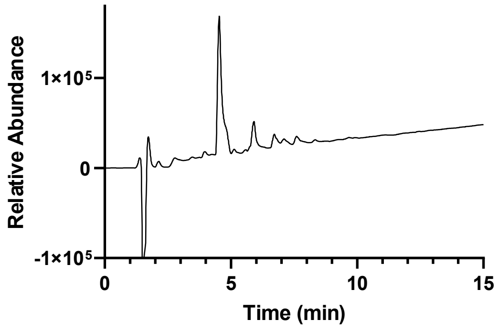

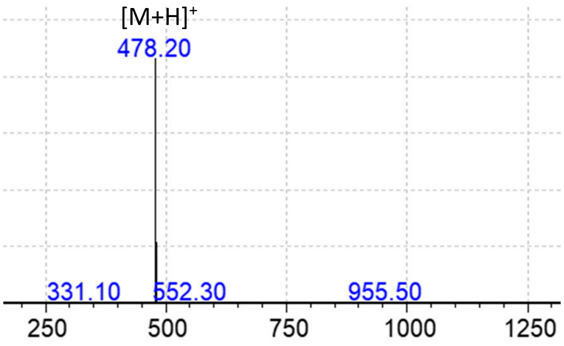

19-FPSAG

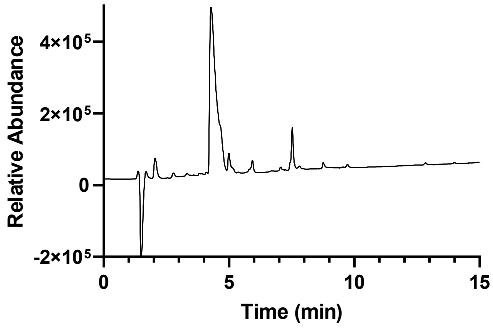

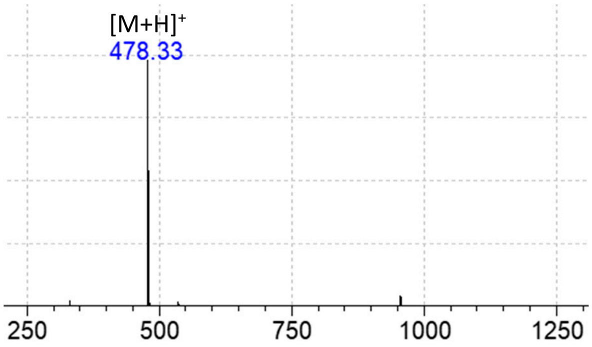

20-FPTAG

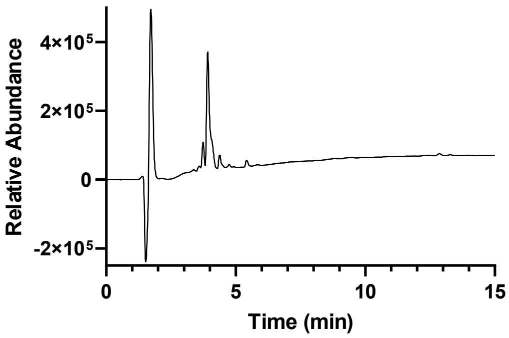

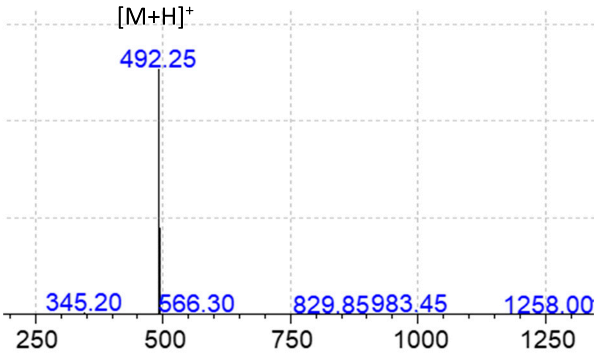

21-FPTAG

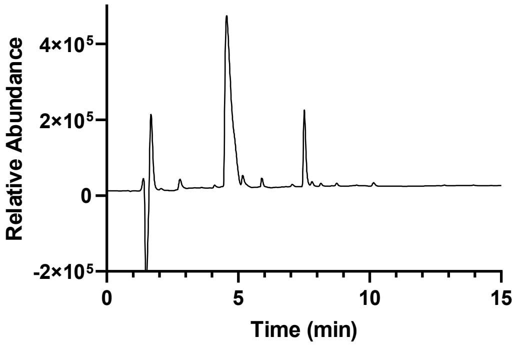

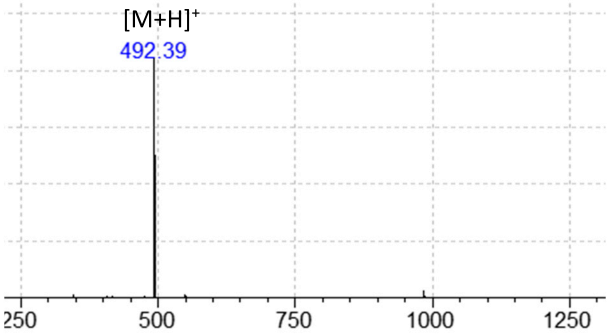

22-FPYAG

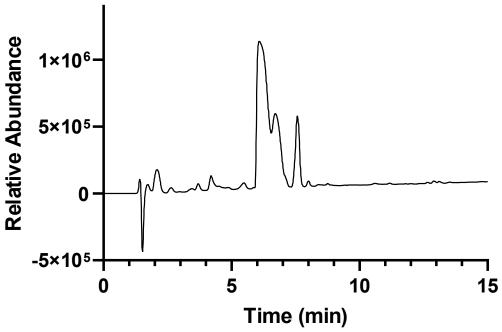

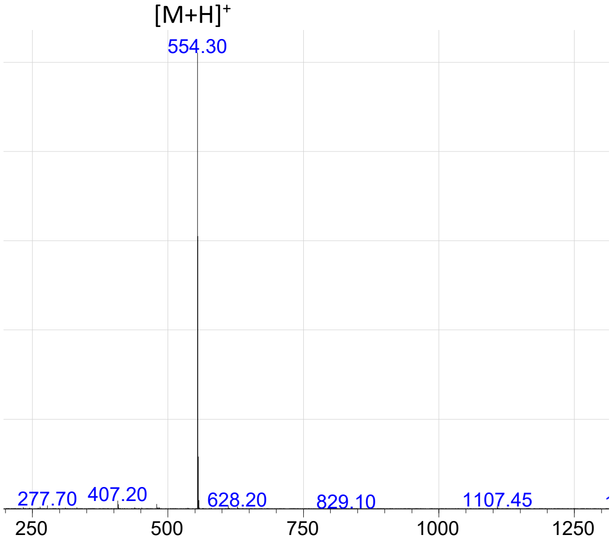

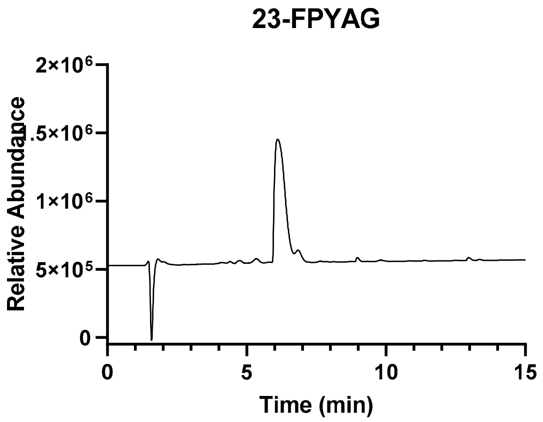
23-FPYAG
Relative Abundance
2×10⁶
1.5×10⁶
1×10⁶
5×10⁵
0
0
5
10
15
Time (min)

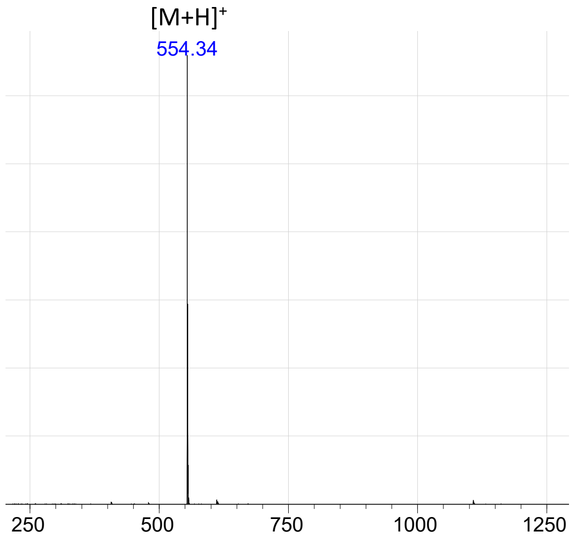
[M+H]⁺
554.34
250
500
750
1000
1250

Table S2

Entry	Peptides[1]	Theoretical Mass (Da)	Experimental Mas (Da)[2]	HPLC retention time (min)[3]	Gradient	Purity[4]
1	FGAFPIAG	778.39	779.40	6.0	5-40%	94%
2	FGAFPVAG	764.38	765.30	9.1	5-40%	91%
3	FGAFPEAG	794.35	795.41	7.9	5-40%	97%
4	GAFGAFPVAG	892.43	893.35	10	5-40%	90%
5	EAFGAFPEAG	994.43	995.31	8.5	5-40%	89%
6	GDEAFGAFPEAG	1166.48	1167.47	9	5-40%	80%
7	FGAFGAFGAFPIAG	1328.64	1330.60	13.5	5-40%	76%
8	DYKG	481.21	482.25	1.6	5-40%	60%
9	DYKG[5]	481.21	482.26	1.6	5-40%	77%
10	DYKGG	538.23	539.25	2.8	0-30%	62%
11	DYKGG[5]	538.23	539.36	1.6	5-40%	72%
12	DYKD	539.21	540.25	3.8	0-40%	63%
13	DYKDD	654.24	655.20	4.9	0-20%	63%
14	DYKK	552.28	553.20	2.1	0-40%	57%

[1] All peptides are C-terminal carboxylic acid; [2] Determined by LC-MS; [3] Linear MeCN (+0.036% TFA) into H_2O (+0.045% TFA) over 15 min; [4] Determined by analytical HPLC; [5] Asp (D) protected as OFm ester; Tyr (Y) protected with 2-Br-Z.

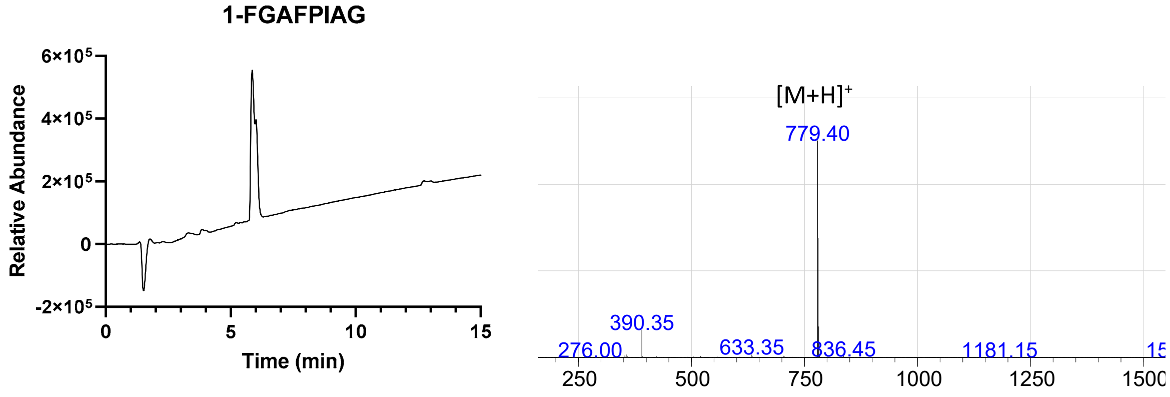

2-FGAFPVAG

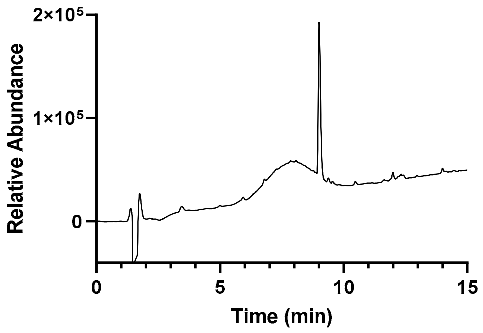

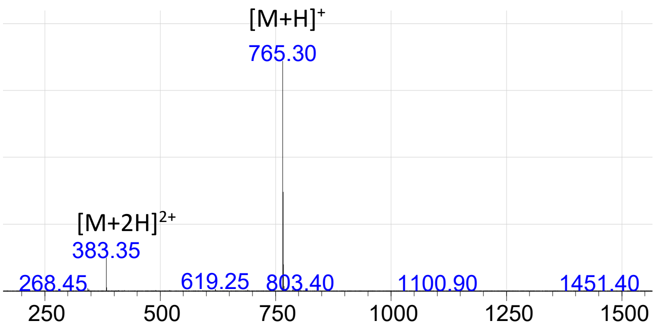

3-FGAFPEAG

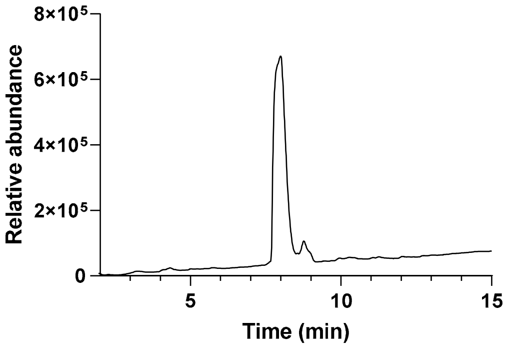

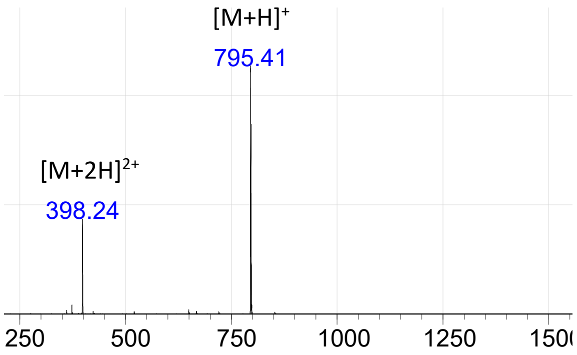

4-GAFGAFPVAG

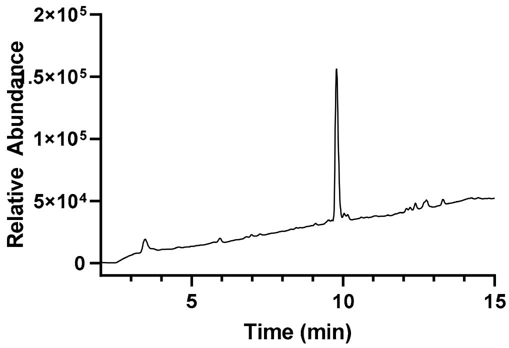

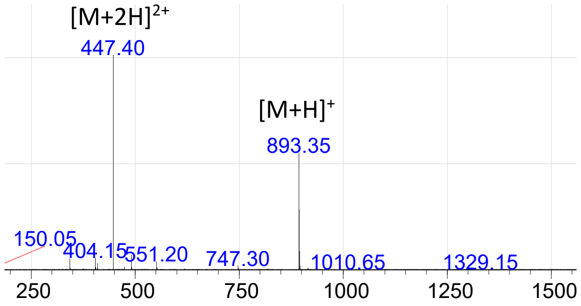

5-EAFGAFPEAG

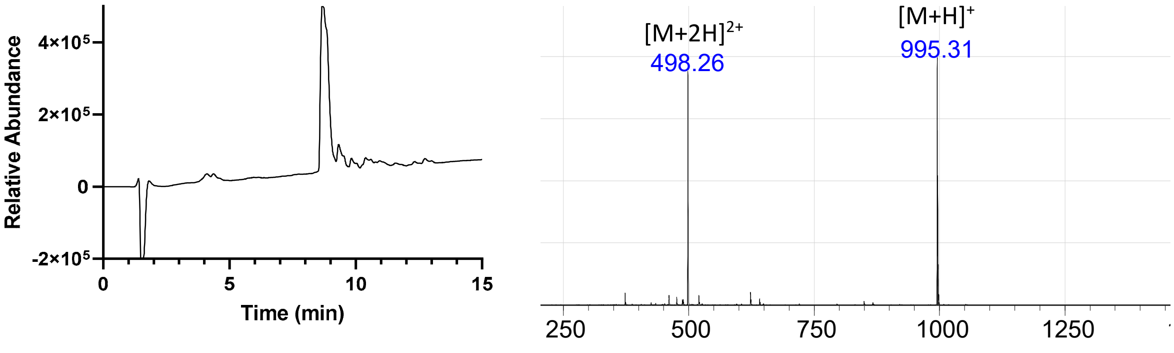

6-GDEAFGAFPEAG

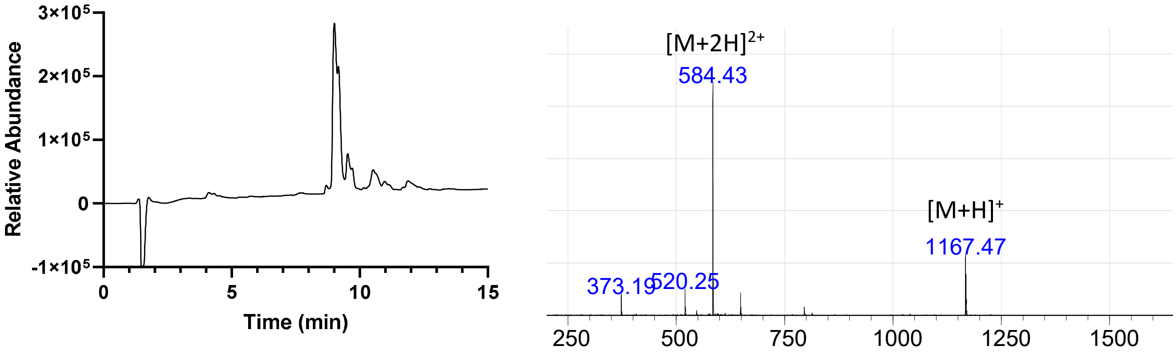

7-FGAFGAFGAFPIAG

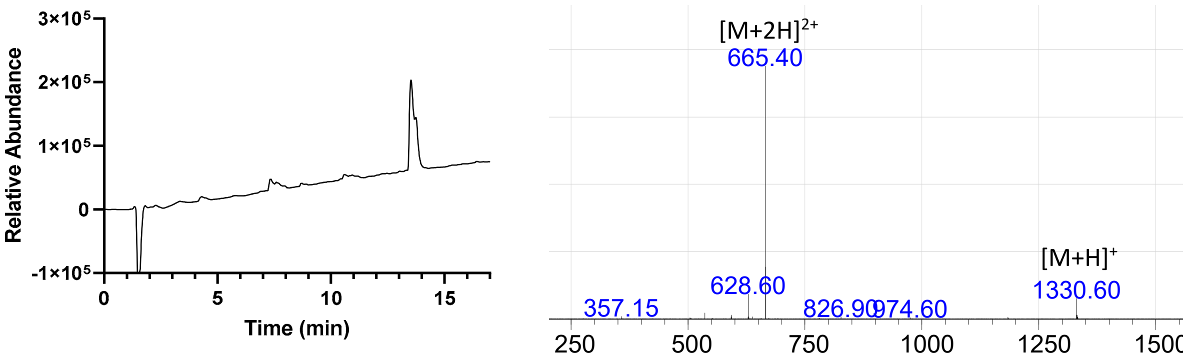

8-DYKG

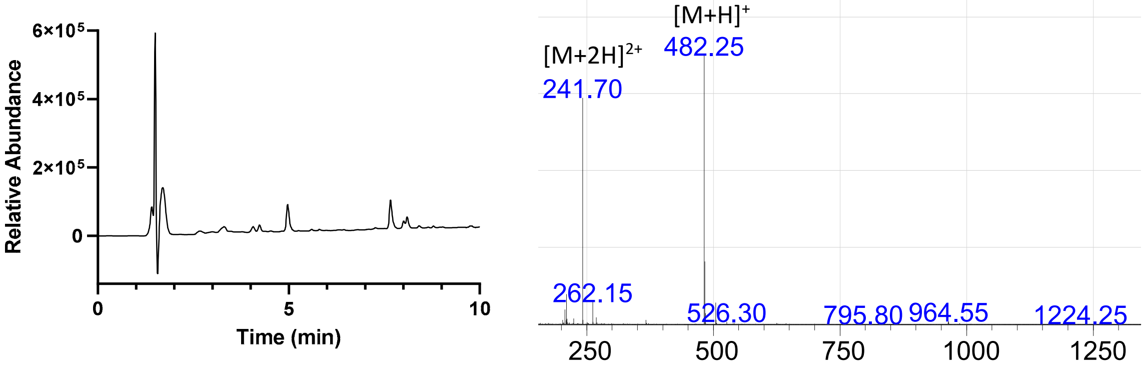

9-DYKG

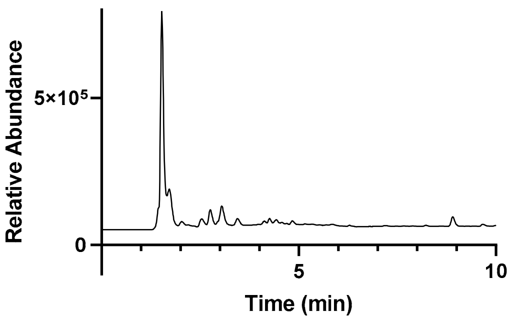

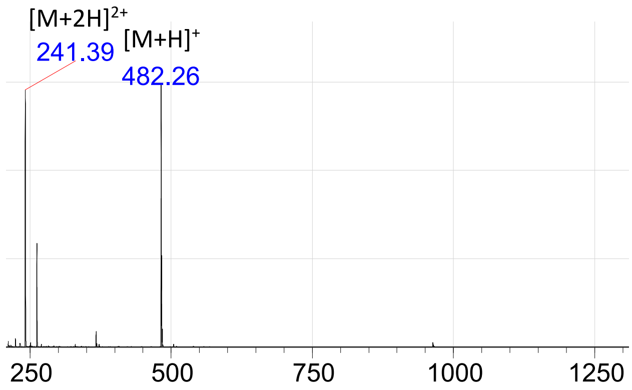

10-DYKGG

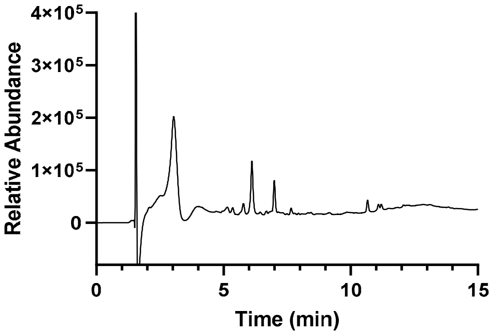

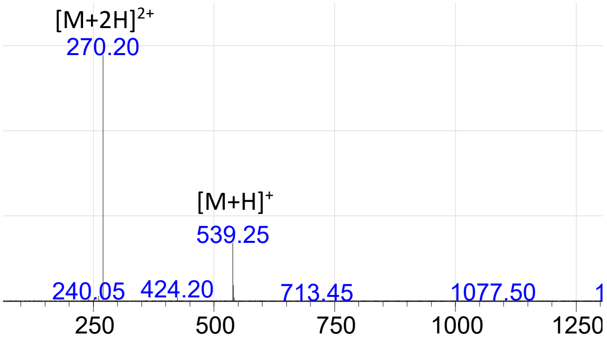

11-DYKGG

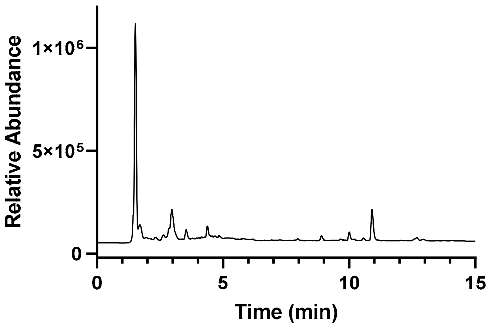

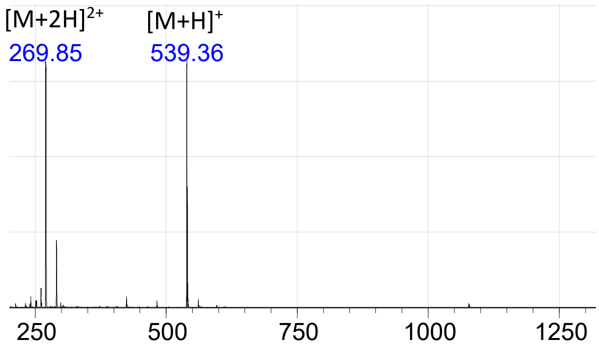

12-DYKD

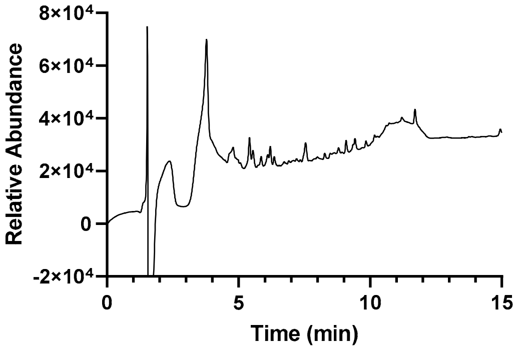

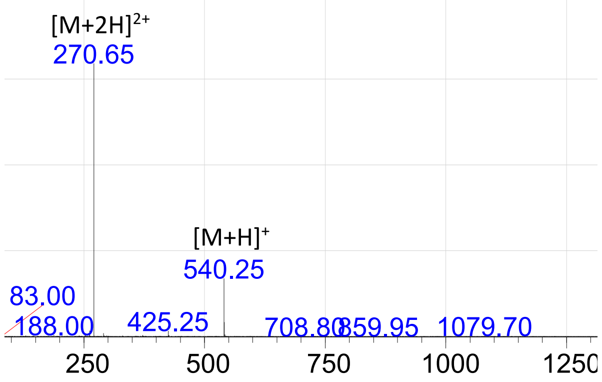

13-DYKDD

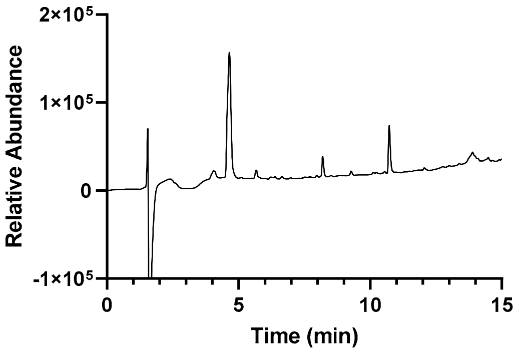

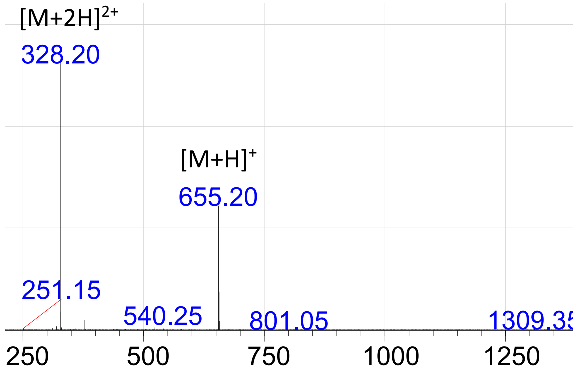

14-DYKK

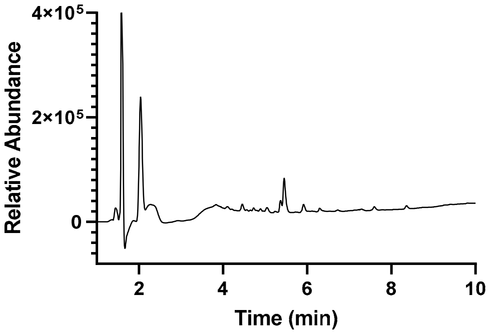

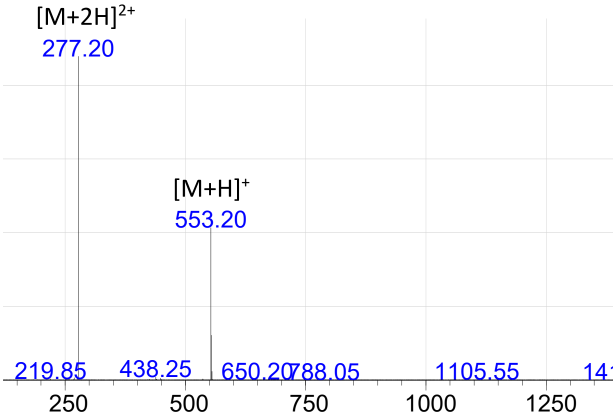

Figure S2

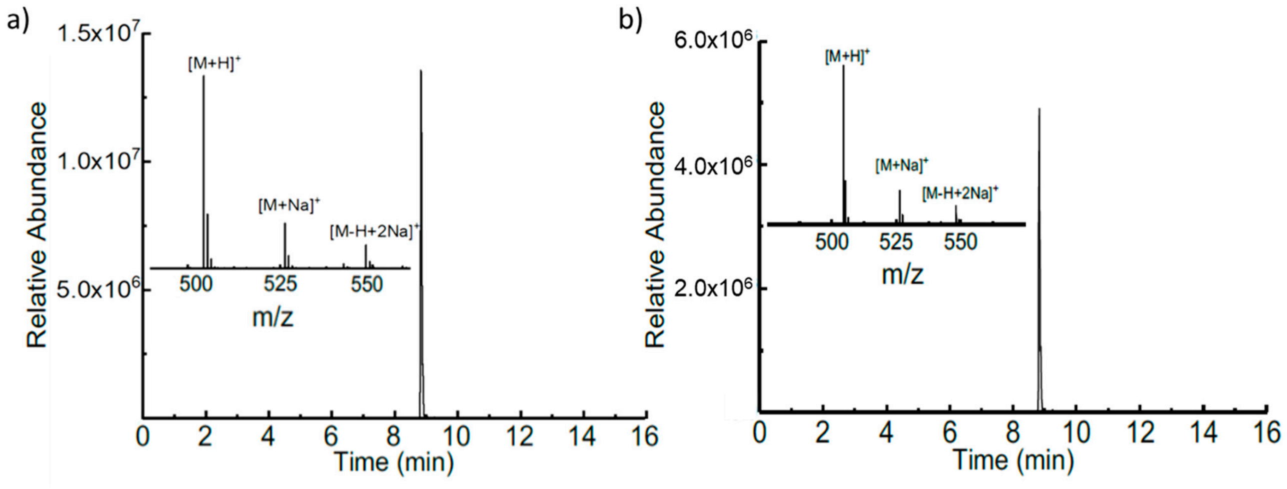

Figure S2: LC-MS of crude FPIAG synthesized on a SPR chip using a) standard cycles and b) fast timing cycles.

Table S3

Amino acid	d_n/d_c (mL/g)	Mass	Volume	d_n/d_c (mol/mL)	n_A-n_C	d_A	moles/cm^2	molecules/cm^2
Gly	0.175	75	91	13.125	0.02864	4.497941	9.81494^{-11}	6.12452^{+13}
Ala	0.167	89	108	14.863	0.02863	4.762203	9.17324^{-11}	5.7241^{+13}
Ile	0.179	131	159	23.449	0.11149	5.417502	2.57579^{-10}	1.60729^{+14}
Pro	0.165	115	139	18.975	0.06024	5.180101	1.64453^{-10}	1.02619^{+14}
Phe	0.244	165	200	40.26	0.24304	5.848035	3.53032^{-10}	2.20292^{+14}

Variations in surface density calculated by the De Feijter equation:

$$\tau = \frac{d_A(n_A - n_c)}{{d_n}/{d_c}}$$

where d_A is the thickness of the adsorbed layer, ${d_n}/{d_c}$ is the refractive index increment of the amino acids as found in literature, n_A and n_c are refractive indexes of adsorbed amino acid layer and the background solution (PBS 0.1X) calculated from experimental data.
